# Dried blood spot sample extraction for metabolomics and proteomics profiling for clinical trials: a descriptive exploratory study

**DOI:** 10.1038/s41598-026-46874-3

**Published:** 2026-04-13

**Authors:** Siri Fägerstam, Emir Johansson, Peder af Geijerstam, Karin Rådholm, Bijar Ghafouri

**Affiliations:** 1 Division of Clinical Medicine, Department of Health, Medicine and Caring Sciences, Linköping University, Linköping, Sweden; 2 Primary Healthcare Center Kärna, Region Östergötland, Linköping, Sweden; 3 Primary Healthcare Center Cityhälsan Centrum, Region Östergötland, Norrköping, Sweden; 4 The George Institute for Global Health, University of New South Wales, Sydney, Australia

**Keywords:** Type 2 diabetes, Biomarkers, Omics, Blood, Biochemistry, Biological techniques, Biomarkers, Diseases

## Abstract

Biomarker research relies on venous blood sampling, which demands trained professionals and proper sample handling, presenting challenges for both rural health care providers and large-scale studies. This study aims to investigate the use of dried blood spots (DBS) as a minimally invasive, patient-centric alternative for biomarker discovery in preparation of a decentralized type 2 diabetes (T2D) trial. To optimize and validate the method, DBS were collected from 2 and 14 healthy participants, respectively. Protein extraction was optimized, evaluated, and samples were analyzed by multi-spot antibody assays, two-dimensional gel electrophoresis, liquid chromatography coupled to tandem mass spectrometry, and nuclear magnetic resonance spectroscopy. The main findings revealed adequate extraction from filter paper, with detection of low abundant inflammatory cytokines. Additionally, proteins and metabolites previously implicated in T2D pathophysiology were detected. In conclusion, the findings in this study support DBS-based proteomics and metabolomics for the identification of novel biomarkers associated with human disease, which offers a scalable and accessible alternative to conventional blood sampling methods for clinical trial studies.

**Supplementary Information:**

The online version contains supplementary material available at 10.1038/s41598-026-46874-3.

## Introduction

A valuable tool in the management and prevention of disease is the use of blood biomarkers; not only for screening but also for diagnostic and prognostic purposes^1^. A major drawback in biomarker screening and analysis of blood is the sampling and transport to laboratories. The blood needs to be drawn by trained personnel in medical facilities, processed to extract the plasma or serum, and transported within 24 h in cold conditions^2–4^. This can be avoided by using dried blood spots (DBS) as sampling method for biomarker discovery in both proteomic- and metabolomic methods^5^. Several studies on the stability of DBS in storage conditions have been done^6–8^, with one study showing stability of up to a month with samples stored in + 40 °C^8^. Most studies, however, suggest that long-term storage of DBS samples should be in at least − 20 °C to ensure stability and minimize biomarker degradation. Furthermore, the use of DBS is patient-centric, as it involves a less invasive procedure that only requires a small amount of blood. It can also be performed by patients themselves in the comfort of their homes, obtained via a small finger prick using a lancet^9,10^. Using home-sampling instead of blood sampling at medical facilities also enables inclusion of participants in rural areas, who would otherwise not be able to participate in interventional studies dependent on blood samples^11^.

Common biomarkers used clinically today to evaluate glycemic status and blood lipids include glycated hemoglobin A_1c_^12,13^, triglycerides, and cholesterol (including total, high-density lipoprotein (HDL), low-density lipoprotein (LDL), and non-lipid cholesterol)^14^. Besides these, biomarkers related to systemic inflammation are associated with T2D as well as cardiovascular disease^15^. These include, among others, C-reactive protein (CRP), interleukin 6 (IL-6), and tumor necrosis factor (TNF)^15–17^. In T2D, inflammation can act as a trigger for insulin resistance and β-cell dysfunction, and proinflammatory markers such as CRP, IL-6, and TNF can therefore be used as potential biomarkers for the development and progression of T2D^18–20^.

For biomarker discovery, two main fields are regularly utilized: proteomics and metabolomics^21,22^, both of which have been applied in biomarker discovery using serum and plasma from patients with T2D. For example, previous research has used two-dimensional gel electrophoresis (2-DE) to investigate differences in T2D proteome profiles and thereby identify biomarkers^23^. Other research has utilized liquid chromatography-mass spectrometry (LC–MS/MS) for proteomic identification of kidney injury biomarkers in T2D^24^, as well as for metabolomic identification of biomarkers and exploring potential T2D treatment targets^25^. Furthermore, nuclear magnetic resonance (NMR) spectroscopy has been used for metabolomic identification of markers associated with endothelial dysfunction, an initial feature in early onset T2D^26^. Recently we have published a study protocol for a decentralized randomized clinical trial where we plan to use DBS to analyze already known biomarkers, as well as unknown biomarkers using omics analyses to evaluate low-grade inflammation and changes in dietary intake as exploratory outcomes^27^.

Although the use of DBS for sampling of blood for biomarker analysis has been investigated for multiple different analytes^8,28^ using both LC–MS/MS^6,29^ and NMR^30^, most of these DBS extraction protocols have been tailored to either method. Few studies have integrated both proteomic and metabolomic approaches using DBS. We therefore aimed to optimize the extraction protocol of DBS for both targeted and untargeted proteomic and metabolomic analyses using multiplexed antibody-based methods, 2-DE, LC–MS/MS, and NMR.

## Results

### Optimization of protein extraction

#### Examination of suitable extraction buffer

**Fig. 1:**
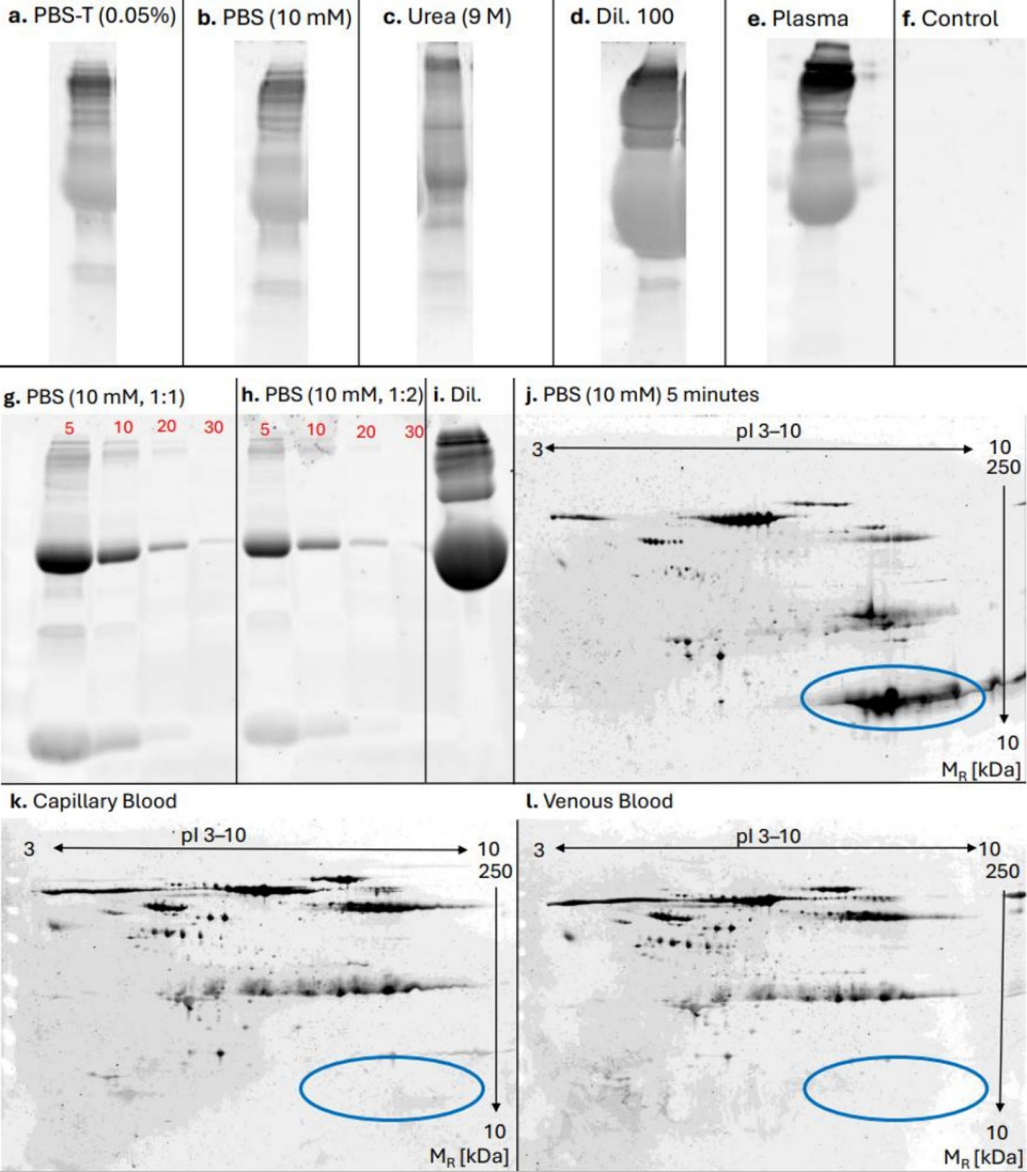
Evaluation of extraction time and proteome. Evaluation of extraction buffer. Gel electrophoresis of dried blood spots extracted at 750 rpm for 20 min using 400 μL of (**a)** PBS-T (0.05%), (**b**) PBS (10 mM), (**c**) urea buffer (9 M), and (**d**) Diluent 100 (Meso Scale Discovery). (**e**) Pooled human plasma was used as positive control, and (**f**) an empty blood spot extracted in PBS-T (0.05%) was used as negative control. n = 1. Gel electrophoresis of dried blood spots extracted at 750 rpm for 5-, 10-, 20-, and 30-min using PBS with (**g)** no dilution and (**h**) twofold dilution. (**i**) Gel electrophoresis of Diluent 100 (Meso Scale Discovery). (**j**) 2-D gel electrophoresis representing the proteome of dried blood spot. (**k**) 2-D gel electrophoresis representing the proteome of capillary blood. (**l**) 2-D gel electrophoresis representing the proteome of capillary blood. pI = Isoelectric point, M_R_ = relative molecular mass. Uncropped gel image is provided in Supplementary Figure S1. n = 1.

To identify the optimal buffer for protein extraction of DBS, four buffer solutions (PBS, PBS-T, UREA, antibody diluent (Diluent 100, Meso Scale Discovery, Rockville, MD, USA) were evaluated. As seen in Fig. 1, sodium dodecyl poly acrylamide gel electrophoresis (SDS-PAGE) revealed higher amounts of low molecular proteins when using PBS (Fig. 1A,B) compared to PBS-T although similar total protein concentrations were measured. Urea buffer resulted in less separation (Fig. 1C) but had similar total protein concentration as PBS and PBS-T. Extraction with Diluent 100 yielded higher overall protein intensity (Fig. 1D). Total protein concentration after using the different buffers is provided in Supplementary Table S1. Pooled human plasma was included as a positive control (Fig. 1E). An empty DBS spot extracted with PBS-T (0.05%) served as negative control, showing no detectable protein bands (Fig. 1F).

#### Examination of extraction time needed using PBS

To evaluate the optimal extraction time, a single DBS was extracted using PBS during the time points 5, 10, 20, and 30 min, as seen in Fig. 1. SDS-PAGE analysis was performed on both undiluted and twofold diluted extracts from each time point (Fig. 1G–H). Efficient protein recovery was observed at 5 min, while prolonged extraction beyond 10 min resulted in a reduction of low molecular weight proteins (Fig. 1G). To assess the purity of Diluent 100, SDS-PAGE was done on the buffer alone, revealing substantial protein content in the absence of blood (Fig. 1I). Quantitative protein concentrations for all time points are provided in Supplementary Table S2.

#### Evaluation of proteome extracted with PBS for 5 min using 2-DE

To assess the proteome composition of proteins extracted from DBS, 2-DE was performed (Fig. 1J). The resulting protein patterns exhibited clear separation across both the isoelectric focusing and SDS-PAGE dimensions. Comparative analysis with reference 2-DE profiles from capillary and venous blood samples in the laboratory’s gel database revealed a similar distribution pattern (Fig. 1K–L), indicating that the extracted proteins reflect a representative blood proteome. High amount of hemoglobulin was detected in DBS samples (marked area Fig. 1).

#### Evaluation of proteome using targeted multi-spot antibody assay

To examine the compatibility of the extraction protocol with targeted proteomics, multi-spot antibody assays were conducted to quantify inflammatory cytokines (IL-1β, IL-4, IL-6, IL-8, IL-10, TNF) and CRP. DBS samples were extracted using PBS for 5, 10, 20, and 30 min, with both undiluted and twofold dilution for cytokine analysis. For CRP quantification, extracts were tested at undiluted, 500-fold and 1000-fold dilutions.

**Fig. 2:**
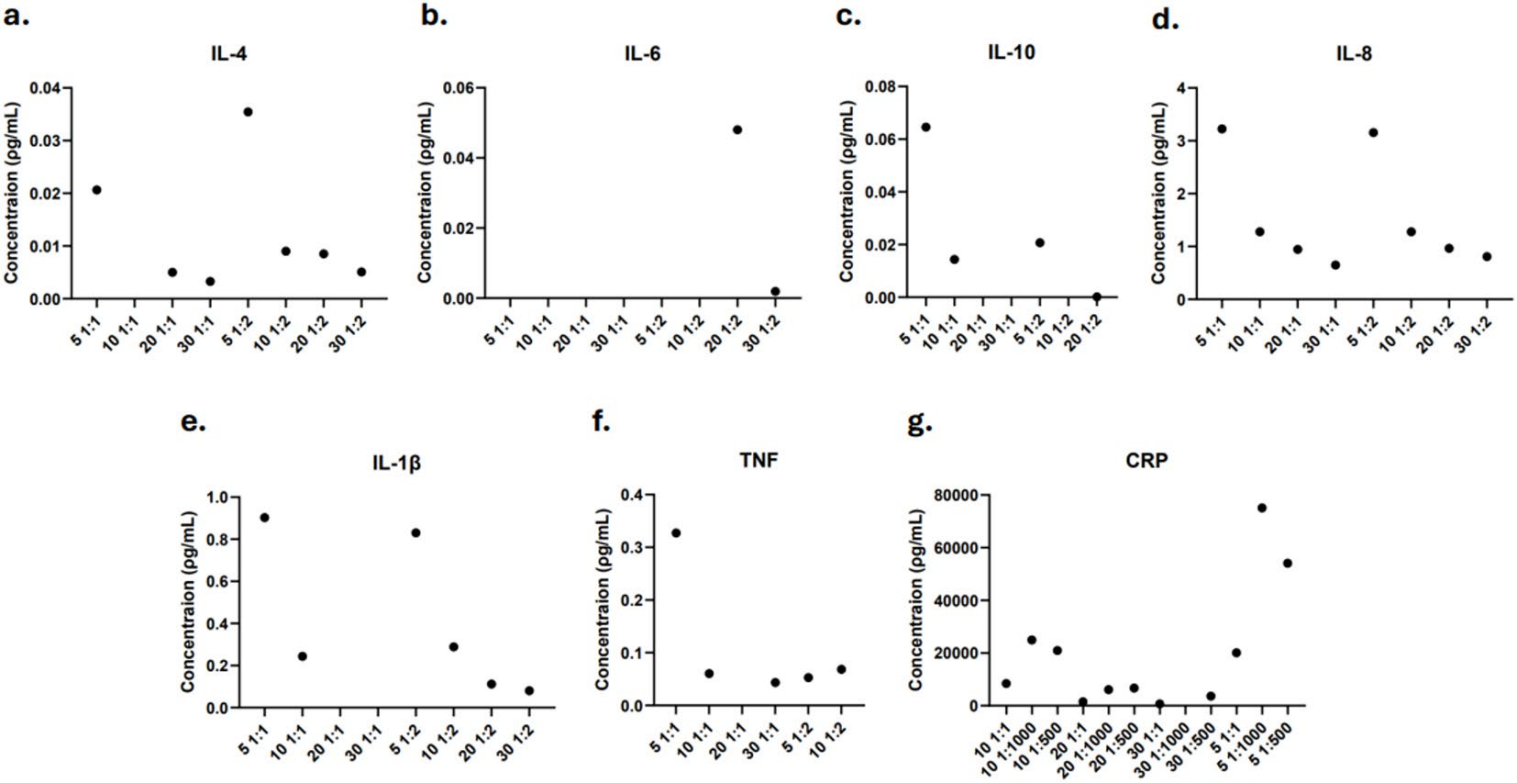
Protein concentration of proinflammatory cytokines and CRP from extracted DBS. (**a**) Concentration of IL-4, (**b**) IL-6, (**c**) IL-8, (**d**) IL-10, (**e**) IL-1β, and (**f**) TNF from extraction time points 5, 10, 20, and 30 min with no dilution and twofold dilution. (**g**) Concentration of CRP from extraction time points 5, 10, 20, and 30 min with no dilution and 500- and 1000-fold dilution. y-axis shows concentration (ρg/mL), x-axis indicates sample, n = 1.

As shown in Fig. 2A–F, all cytokines except IL-6 were detectable in extracts obtained after 5 and 10 min without dilution, and after 5 min with twofold dilution. CRP concentrations were measurable at all time points except 30 min (Fig. 2G), with the highest levels observed following 5-min extraction at 500-fold and 1000-fold dilutions. Detailed concentrations for cytokines and CRP are provided in Supplementary Table S3 and Table S4, respectively.

### Validation of protocol by application on research subjects

#### Total protein concentration between DBS circles and individuals

**Fig. 3:**
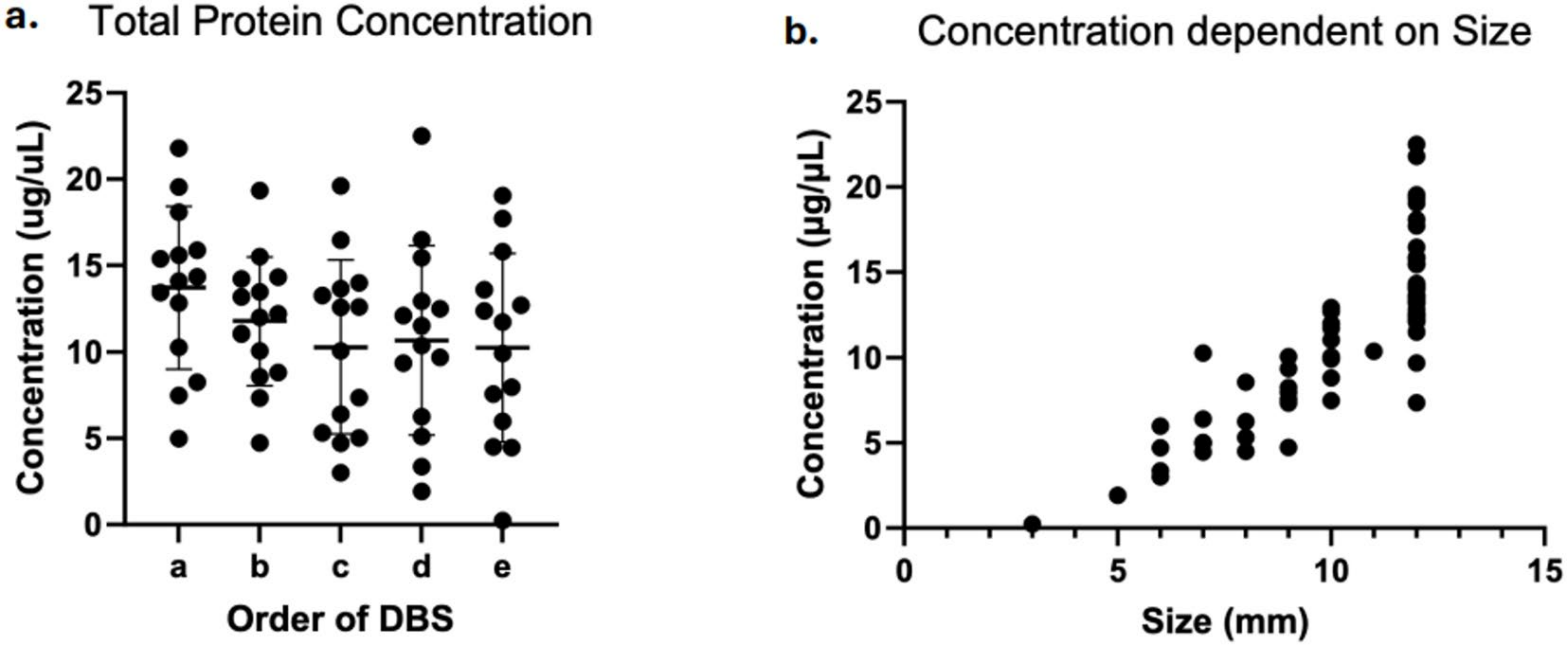
Total protein concentration of DBS between individuals and circles. (**a**) Protein concentrations measured using the 2-D Quant Kit. Bars indicate concentration between research subjects and between circles from one individual DBS card (**a**–**e**). y-axis shows concentration (μg/mL), x-axis indicates sample. Data is presented as mean ± SD. SD for a = 4.703, b = 3.726, c = 5.033, d = 5.481, and e = 5.444 (μg/μL). (**b**) Protein concentration between different sizes of DBS circles. Y-axis shows concentration (μg/mL), x-axis shows size of circles (mm). n = 14.

To evaluate inter- and intra-individual variability in protein yield, total protein concentrations were measured from five sequential circles (a–e) collected on the filter paper (Fig. 3A). Variations in protein concentration were observed both between individuals and among DBS from the same individual. As illustrated in Fig. 3B, protein yield had a size-dependent pattern, with larger diameters associated with higher concentrations. Specifically, DBS measuring 10–12 mm in diameter consistently yielded protein concentrations exceeding 10 μg/mL. Across all samples, protein concentrations ranged from 0.23 to 22.5 μg/mL. A comprehensive list of measured concentrations is provided in Supplementary Table S4.

#### Evaluation of targeted proteome between individuals using targeted multi-spot antibody assay

**Fig. 4:**
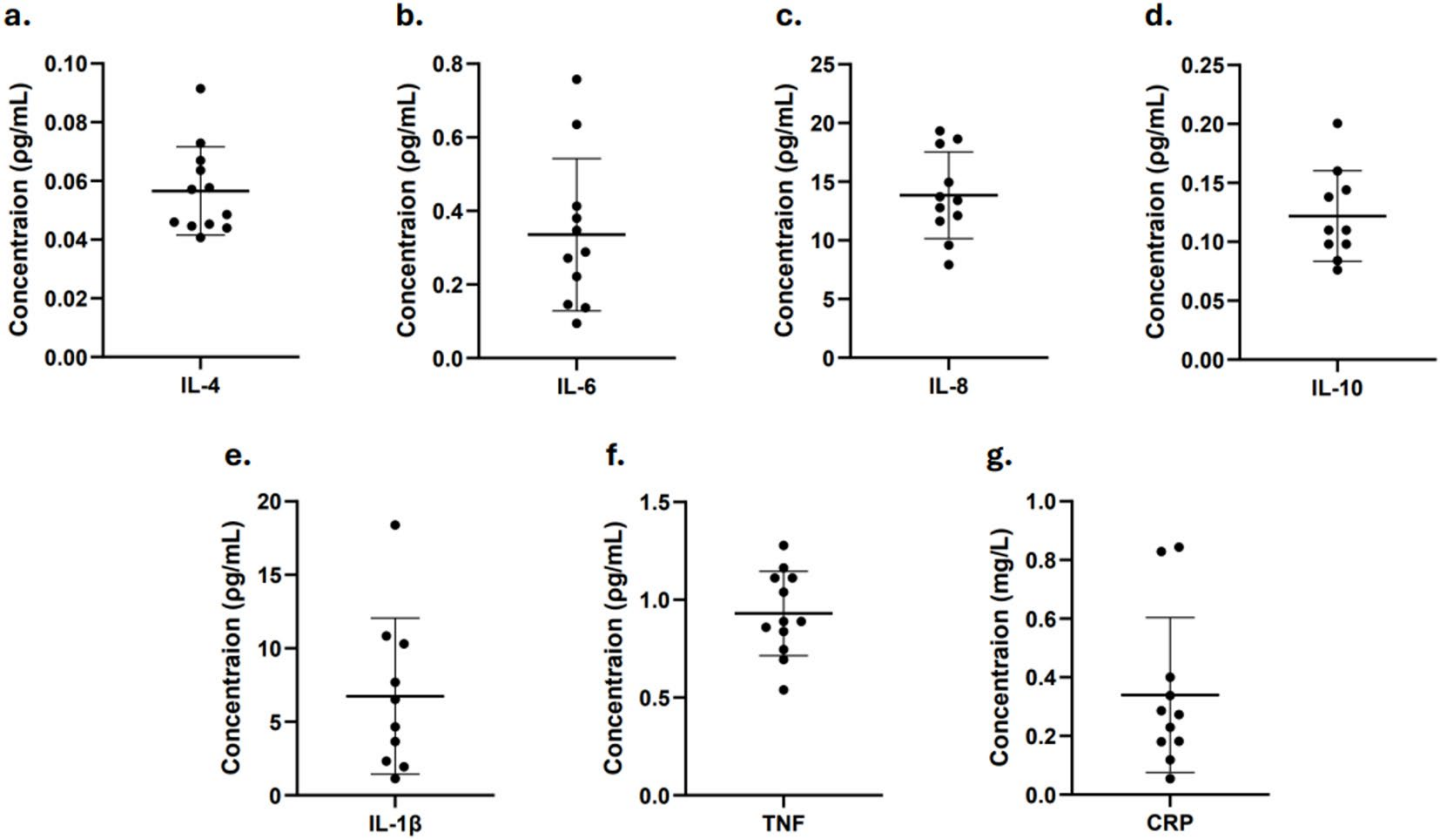
Protein concentration of proinflammatory cytokines and CRP from healthy subjects. Concentration of (**a**) IL-4, (**b**) IL-6, (**c)** IL-8, (**d**) IL-10, (**e**) IL-1β, and (**f**) TNF, extracted for 5 min in PBS with no dilution. (**g**) Concentration of CRP extracted for 5 min in PBS with 500-fold dilution. y-axis shows concentration (pg/mL), x-axis indicates sample. Data is presented as mean ± SD. SD for (**a**) = 0.015 (n = 12), (**b**) = 0.21 (n = 11), **c** = 3.67 (n = 11), (**d**) = 0.038 (n = 10), **e** = 5.31 (n = 10), (**f**) = 0.22 (n = 12), and (**g**) = 0.26 (n = 11) (mg/L).

DBS of same size (12 mm) was used for targeted protein analysis. Quantification of cytokines was successful in all samples, except for IL-10, which was undetectable in two cases (Fig. 4A–F). To adjust for sample dilution during extraction, all reported values were multiplied with a dilution factor of 8 to make it comparable with plasma.

Mean concentration was as follows: 0.057 (± 0.015) ρg/mL for IL-4, 0.34 (± 0.21) ρg/mL for IL-6, 13.84 (± 3.67) ρg/mL for IL-8, 0.12 (± 0.038) ρg/mL for IL-10, 6.75 (± 5.31) ρg/mL for IL-1β, and 0.93 (± 0.22) ρg/mL for TNF. A complete dataset of cytokine concentrations is provided in Supplementary Table S6. Among the measured cytokines, IL-1β and IL-8 exhibited the highest concentrations overall, with IL-8 being the most abundant in most samples (Fig. 4A–F). CRP concentrations were relatively consistent across samples, with a mean concentration of 0.34 (± 0.26) mg/L (Fig. 4G). A complete dataset of CRP concentrations is provided in Supplementary Table S7. Outlier values were identified using the recommended method (ROUT) in GraphPad Prism 10 based on False Discovery Rate and Q set to 1%. For IL-6, IL-8, and CRP one outlier each was identified and for IL-1β and IL-10 two outliers each were identified. These outliers were excluded from subsequent descriptive statistical analyses. Comparison of descriptive statistical analyses with and without outlier exclusion is provided in Supplementary Table S8.

#### Untargeted proteomics using liquid chromatography-mass spectrometry

**Fig. 5:**
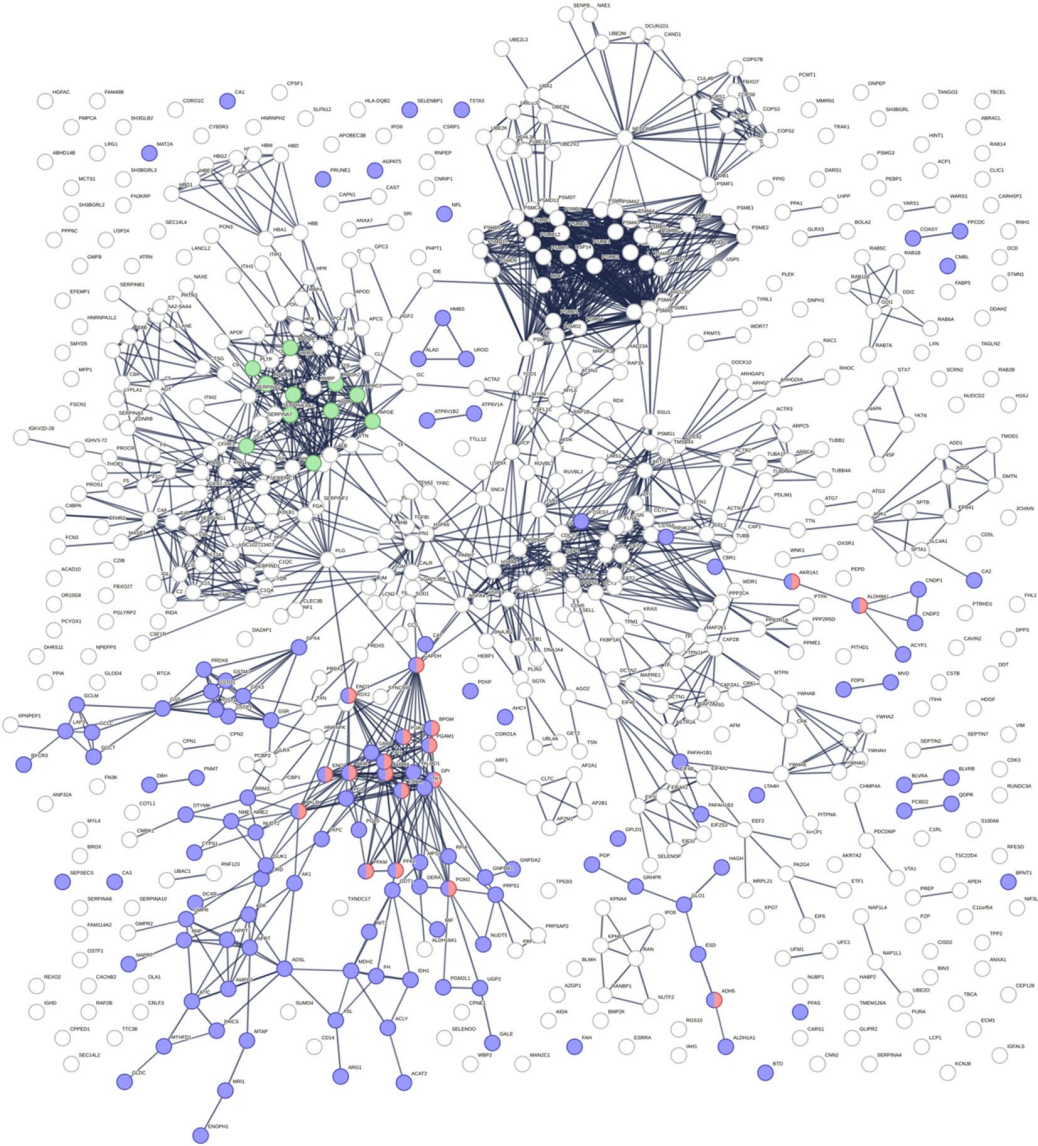
STRING network of proteins identified in LC–MS/MS. Purple nodes represent metabolic pathways, pink nodes represent glycolysis/gluconeogenesis, and green nodes represent cholesterol metabolism. Each node represents a protein identified in the LC–MS/MS analysis, and lines indicate interactions between individual proteins. PPI enrichment* p*-value < 1.0 e−16, n = 5.

LC–MS/MS analysis was performed on five DBS samples of similar total protein concentrations resulting in the identification of 765 unique proteins. Proteins detected in less than 60% of samples were excluded from further analysis (n = 11), yielding a dataset of 754 proteins. For each protein, mean, standard deviation, and coefficient of variation (CV) were calculated. Based on the small sample size, proteins with CV < 10% were kept to avoid a large variation, leading to the exclusion of three proteins and a final set of 751 proteins (Fig. 5). The full list of proteins is provided in Supplementary Table S9.

Bioinformatic analysis using KEGG pathway mapping^31–33^ revealed enrichment in metabolic pathways (purple nodes), glycolysis/gluconeogenesis (pink nodes), and cholesterol metabolism (green nodes) (Fig. 5). In addition to a high abundance of immunoglobulins, proteins involved in glucose synthesis, lipid metabolism and transport, and inflammation were identified. A selection of proteins related to these processes and involved in the pathogenesis of T2D are presented in Table 1. Notably, insulin-like proteins, vitamin D-binding protein, angiotensinogen, and hemoglobin were detected (Table 1).

**Table 1:** Identified and quantified proteins of DBS by LC–MS/MS.

Accession number	Protein	Gene	Mean intensity (n = 5)	SD	CV (%)
P11413	Glucose-6-phosphate 1-dehydrogenase	G6PD	711.65	26.35	0.37
P32119	Peroxiredoxin-2	PRDX2	37,701.20	1646.99	0.44
P52209	6-phosphogluconate dehydrogenase, decarboxylating	PGD	1044.80	46.25	0.44
P48506	Glutamate–cysteine ligase catalytic subunit	GCLC	641.82	28.82	0.45
P14735	Insulin-degrading enzyme	IDE	148.72	8.84	0.59
P01024	Complement C3	C3	7249.92	437.72	0.60
P02774	Vitamin D-binding protein	GC	1266.40	77.83	0.61
P08697	Alpha-2-antiplasmin	SERPINF2	917.07	56.67	0.62
P00338	L-lactate dehydrogenase A chain	LDHA	636.45	42.09	0.66
P00915	Carbonic anhydrase 1	CA1	38,999.35	2736.92	0.70
P06733	Alpha-enolase	ENO1	1609.77	118.77	0.74
P01031	Complement C5	C5	642.54	51.18	0.80
P04406	Glyceraldehyde-3-phosphate dehydrogenase	GAPDH	6853.19	546.14	0.80
P06727	Apolipoprotein A-IV	APOA4	3880.65	327.55	0.84
P14618	Pyruvate kinase	PKM	212.61	17.95	0.84
P69905	Hemoglobin subunit alpha	HBA1	121,999.86	11,164.66	0.92
P05156	Complement factor I	CFI	901.25	85.67	0.95
P02652	Apolipoprotein A-II	APOA2	5275.80	501.72	0.95
B4E1Z4	Complement factor B	-	2510.34	263.90	1.05
P01019	Angiotensinogen	AGT	1792.10	207.23	1.16
P06681	Complement C2	C2	264.24	31.78	1.20
P02647	Apolipoprotein A-I	APOA1	54,291.57	6895.07	1.27
P35858	Insulin-like growth factor-binding protein complex acid labile subunit	IGFALS	605.45	80.49	1.33
P02748	Insulin-like growth factor 2	IGF2	92.12	17.73	1.92
Q9GZN8	Adipose-secreted signaling protein	ADISSP	167.54	53.31	3.18
Q01469	Fatty acid-binding protein 5	FABP5	33.29	13.57	4.08

List of proteins related to T2D pathogenesis. Accession numbers retrieved from the UniProt protein database. Protein name, gene, mean intensity, standard deviation and coefficient of variation are presented. n = 5.

#### Untargeted metabolomics using nuclear magnetic resonance spectroscopy

NMR analysis was conducted on all 14 DBS samples from DBS circles of similar total protein concentrations to evaluate the compatibility of a metabolomic profiling method. After analysis, 112 metabolites were detected, and the same exclusion criteria as previously described were applied, resulting in a final dataset of 67 metabolites. Of these, 32 metabolites were within the range of the model provided by the manufacturer (Bruker Daltonics, Bremen, Germany) (Table 2). The full list of metabolites is provided in Supplementary Table S10.

**Table 2:** Quantification of lipid metabolites in DBS identified by NMR.

Abbreviation	Explanation	Mean concentration (n = 14)	SD	CV (%)	95% Range of Model
L1TG	LDL1-Triglycerides	6.32 [mg/dL]	0.08	0.13	3–14
H3TG	HDL3-Triglycerides	1,13 [mg/dL]	0.06	0.52	1–5
ABA1	Apo-B / Apo-A1	0.95 [–/–]	0.10	1.08	0.30–1.07
VLAB	VLDL-Apo-B	3.81 [mg/dL]	0.42	1.11	3–26
VLPN	VLDL- Particle number	69.29 [nmol/L]	7.71	1.11	50–473
L1PL	LDL1-Phospholipids	9.35 [mg/dL]	1.07	1.15	6–30
L3TG	LDL3-Triglycerides	2.18 [mg/dL]	0.27	1.25	1–6
L1PN	LDL1-particle number	198.02 [nmol/L]	25.41	1.28	98–567
L1AB	LDL1-ApoB	10.89 [mg/dL]	1.40	1.28	5–31
V4TG	VLDL4-Triglycerides	3.34 [mg/dL]	0.48	1.43	3–28
L2AB	LDL2-ApoB	19.71 [mg/dL]	3.40	1.72	3–23
L1CH	LDL1-Cholesterol	15.45 [mg/dL]	2.70	1.75	8–59
L2PL	LDL2-Phospholipids	17.67 [mg/dL]	3.15	1.78	2–25
L2TG	LDL2-Triglycerides	3.39 [mg/dL]	0.61	1.79	1–6
L3FC	LDL3-Free Cholesterol	4.18 [mg/dL]	0.76	1.83	1–13
L2CH	LDL2-Cholesterol	34.76 [mg/dL]	6.76	1.94	2–48
H2TG	HDL2-Triglycerides	1.15 [mg/dL]	0.23	1.97	1–5
L3PL	LDL3-Phospholipids	9.09 [mg/dL]	1.82	2.01	2–24
L3AB	LDL3-ApoB	10.21 [mg/dL]	2.28	2.23	3–27
L3PN	LDL3-particle number	185.62 [nmol/L]	41.48	2.23	51–499
L2FC	LDL2-Free Cholesterol	7.88 [mg/dL]	1.88	2.38	1–14
V2FC	VLDL2- Free Cholesterol	0.15 [mg/dL]	0.04	2.42	0–7
L3CH	LDL3-Cholesterol	16.66 [mg/dL]	4.24	2.55	3–46
H1TG	HDL1-Triglycerides	2.86 [mg/dL]	0.77	2.69	1–12
V1TG	VLDL1-Triglycerides	8.80 [mg/dL]	2.40	2.73	6–212
L1FC	LDL1-Free Cholesterol	3.10 [mg/dL]	0.89	2.88	2–17
LDHD	LDL-cholesterol / HDL-cholesterol	3.55 [–/–]	1.18	3.32	0.98–4.08
V5FC	VLDL5- Free Cholesterol	0.90 [mg/dL]	0.44	4.93	0–2
V5PL	VLDL5-Phospholipids	1.50 [mg/dL]	0.77	5.13	0–5
V5TG	VLDL5-Triglycerides	1.80 [mg/dL]	0.93	5.18	1–7
H1CH	HDL1-Cholesterol	6.64 [mg/dL]	3.74	5.64	6–46
V5CH	VLDL2-Cholesterol	1.21 [mg/dL]	0.75	6.21	0–4

List of lipid subfractions within the 95% range of model and CV < 10%. Metabolite abbreviation, explanation, mean concentration, standard deviation and coefficient of variation are presented. HDL = high density lipoprotein, LDL = low-density lipoprotein, VLDL = very low-density lipoprotein, Apo-A = apolipoprotein A, Apo-B = apolipoprotein B100. n = 14.

**Fig. 6:**
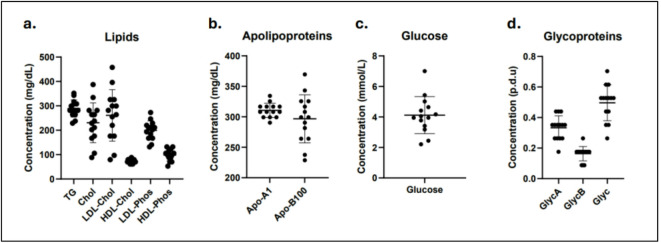
Metabolite concentrations from healthy subjects. Concentrations of (**a**) lipids, (**b**) apolipoproteins (**c**), and glycoproteins (**d**) extracted for 5 min using PBS and corrected with a dilution factor of 16. y-axis shows concentrations (mg/dL, mmol/L, and p.d.u), x-axis indicates metabolites. Data is presented as mean ± SD, n = 14.

Concentrations of metabolites related to T2D are illustrated in Fig. 6. To adjust for sample dilution during extraction and analysis, all reported values were multiplied with a dilution factor of 16 to make it comparable with plasma. For lipid components, mean concentrations were: 289.1 (± 35.34) mg/dL for triglycerides (TG), 230.7 (± 81.77) mg/dL for cholesterol (Chol), 260.9 (± 105.5) mg/dL for LDL-Chol, 72.91 (± 8.04) mg/dL for HDL-Chol, 199.3 (± 38.84) mg/dL for LDL-phospholipids (-Phos), and 98.06 (± 23.37) mg/dL for HDL-Phos (Fig. 6A).Within apolipoproteins, mean concentration for Apo-A1 was 310.5 (± 11.67) mg/dL and for Apo-B100 it was 296.7 (± 39.42) mg/dL (Fig. 6B). The mean concentration for glucose was 4.12 (± 1.22) mmol/L (Fig. 6C). For glycoproteins, mean concentrations were 0.33 (± 0.079) procedure defined units (p.d.u) for GlycA, 0.16 (± 0.047) p.d.u for GlycB, and 0.50 (± 0.12) p.d.u for Glyc (Fig. 6D).

## Discussion

The main results of this study indicate that using DBS on filter paper as sampling method is feasible and compatible with both proteomic (targeted and untargeted) and metabolomic analyses. Optimization of the extraction protocol identified PBS as the most suitable extraction buffer, confirmed by the use of PBS-based extraction buffers for DBS in previous protocols^34,35^. While aqueous solutions are generally more suitable for proteomic analyses and measurements of protein concentrations, organic solvents are preferred for metabolite analysis^5^. In this context, we still opted for PBS for metabolomic analysis, as optimizing an extraction protocol fit for both proteomic and metabolomic analyses from one single DBS would simplify and accelerate the sample preparation process.

Furthermore, an extraction time of 5 min was found to be optimal, as prolonged time (≥ 10 min) may cause degradation of unstable proteins, especially the low abundant cytokines and chemokines^36^. Notably, a single drop of blood yielded high enough protein content to perform 2-DE, resulting in a proteomic profile closely resembling that of conventionally collected blood. However, there was one exception. In the DBS gel there was a presence of small proteins, not present on the plasma gels. We hypothesize that these small proteins could be fractions of hemoglobin, and for future studies the DBS extraction should be cleaned up using commercially available depletion columns prior to analysis. Additionally, proinflammatory cytokines could be measured at very low concentrations, proving a high sensitivity of the method.

Applying the optimized protocol on healthy subjects further supports the use of DBS as a sampling method for proteomic analysis. While some inter- and intra-individual variability in protein concentration was observed, examination of full blood spots (ø 12 mm) overall yielded the highest protein concentration. While there is a gap-of-knowledge in measuring cytokine concentration from DBS of healthy subjects, previous research on plasma and serum concentrations of inflammatory interleukins in healthy individuals has been done. In the current study, all proinflammatory cytokines assessed from whole blood were detectable in healthy individuals and the detected concentrations of some cytokines were in the same range as reported in human plasma or serum from healthy subjects. The mean concentrations of IL-6, IL-8, and IL-10 were in accordance to reported range in plasma/serum^37–39^, while concentrations of IL-1β, IL-4, TNF, and CRP were lower^40–43^. The slightly lower concentrations observed in this study may be attributed to the use of whole blood rather than serum or plasma, as erythrocytes or other cellular debris in whole blood could mask the desired analytes, often present in low abundance^34,44^. Additionally, differences in cytokine levels are highly individual and can depend on various factors such as genetic polymorphism^45^ and environmental and lifestyle factors^46,47^.

Despite generally lower total protein concentrations in DBS compared to serum or plasma concentrations, 2–22 μg/μL vs. 60–80 μg/μL^48^, a considerable amount of proteins could still be detected and identified with LC–MS/MS. In particular, the clustering seen in metabolic pathways and cholesterol metabolism are of interest. T2D is a metabolic disorder, and disruptions in these pathways contribute to the pathophysiology and clinical characteristics such as insulin resistance and hyperglycemia^49^. Although glycolysis and gluconeogenesis are commonly implicated as the major metabolic pathways, amino acid metabolism also plays a significant role and relate to both increased and decreased risk of T2D. Higher circulating levels of alanine, glutamate, lysine, and methionine for example are associated with increased risk, while glutamine and glycine are associated with decreased risk^50^. Another emerging contributor to the pathogenesis of T2D is branched-chain amino acid metabolism, consistently found in elevated levels^51^. Thus, exploring various metabolic pathways on a larger scale with the use of proteomics could lead to the discovery of novel biomarkers.

Cholesterol metabolism is also intricately linked to T2D, with affected individuals often exhibiting an increase in cholesterol synthesis together with a reduction in absorption^52^. Finding disruptions early in the cholesterol metabolism could therefore have valuable implications in both early diagnosis and treatment of T2D. Looking closer on singular proteins identified in the LC–MS/MS analysis, other lipid- and adipose related proteins were identified as well. Apolipoproteins, fatty acid-binding proteins, and adipokines are for example believed to be involved in the pathogenesis of T2D^53–55^. Moreover, elevated levels of LDL cholesterol and lowered levels of HDL cholesterol, resulting in an increased risk for cardiovascular disease, is often seen^56^.

In this study, various lipid components, subfractions, and apolipoproteins could be measured using NMR. Mean concentrations for TG and cholesterol were higher than both clinical reference values^57,58^ and previous research on DBS^59^. For lipoproteins, reference values for concentrations of LDL cholesterol, HDL cholesterol, LDL phospholipids, and HDL phospholipids from DBS are limited, but a previous study using plasma samples measured lower mean concentrations in a control group^60^ than the measured levels in the current study. Apolipoproteins, together with lipids, are the main components of lipoproteins, and as such they are thought to be associated with T2D dyslipidemia through the influence on the HDL/LDL ratio^61^. Apo-A1 is the main apolipoprotein forming the HDL particles^62^, while LDL mainly consists of Apo-B100^63^, and the Apo-B100/Apo-A1 ratio is associated with T2D prevalence^64^. In addition, low levels of Apo-A1 in serum has been linked to increased risk of comorbidities such as cardiovascular autonomic neuropathy^65^. In this study, mean concentration of Apo-A1 and Apo-B100 in whole blood were higher than in previous research on serum in healthy controls^66^.

A diagnostic criteria of T2D is fasting plasma glucose > 6.1 mmol/L (110 mg/dL)^67^. For non-diabetic individuals, a healthy fasting plasma glucose is considered to be between 3.9–5.6 mmol/L (70–100 mg/dL) based on healthy controls in a previous study^68^. These reference values confirm the measured glucose concentration in this study, with a mean concentration of 4.12 mmol/L. Another emerging biomarker specific to NMR-analysis are glycoproteins, in particular GlycA and GlycB. These are composite signals from N-acetyl moieties of circulating acute-phase glycoproteins, used to detect acute-phase and low-grade inflammation^69,70^. GlycA levels, specifically, have been associated with concentrations of IL-6, TNF, and CRP^71^, with one cohort study on T2D patients showing an association between higher GlycA levels and increased risk of microvascular complications^72^. In the current study, levels of GlycA, GlycB and the total glycoprotein signal (Glyc) could be detected from DBS of healthy individuals, something that, to the best of our knowledge, has not been done previously.

A limitation in this study is that the exact volume of the DBS is unknown, thus affecting the real concentrations of the measured analytes. To try and compare the concentrations reported herein, dilution factors were calculated using an estimated blood volume of 50 µL, however the real volumes of the droplets may vary depending on drop diameter^73,74^. Thus, standardization through stamping circles of the same size needs to be implemented for further studies.

Additionally, although not performed in the current study, using hematocrit values for normalization of the results would be beneficial. Hematocrit is the ratio of red blood cells to the total blood volume and varies between 25–55% in different individuals^75^, thus affecting the final quantification if not taken into consideration. However, a key advantage of DBS is its suitability for remote clinical trials, where venous blood collection at medical facilities can be avoided, making the determination of hematocrit values impossible. Normalization by total protein concentration after extraction of the DBS is therefore more suitable. Although this was done in the current study for LC–MS/MS- and NMR analyses, it was not done for the multi-spot antibody assays, but the same size of DBS was used.

Another limitation is that PBS was used for extraction of lipoproteins and metabolites in the NMR analysis, organic solvents like methanol will improve the recovery yield of the extraction. Nevertheless, finding an extraction protocol fitting for both proteomic and metabolomic analysis is preferable. The use of NMR for metabolomics is also a matter of discussion. Although LC–MS/MS analysis is advantageous in the number of analytes identified, NMR performs better when it comes to identification and quantification of lipoprotein subclasses and lipids^76^. This is due to the nature of the different analyses, NMR is non-destructive and the lipoprotein as a whole is analyzed. LC–MS/MS on the other hand relies on lipid extraction from the lipoprotein, and the resulting pool of lipid molecules makes it difficult to discern the lipoprotein origin^76,77^.

Furthermore, the current study was solely performed on healthy individuals, limiting generalizability to clinical populations. Thus, the findings in this study need to be validated in populations with comorbidities, including T2D. In such comparative omics studies, it’s highly recommended that normalized values that consider the size of DBS are used. Future studies would benefit from expansion to populations of individuals with T2D to validate the clinical relevance of the identified proteins and pathways, and standardization and automation of the DBS extraction and processing to ensure reproducibility and facilitate high-throughput screening of potential novel biomarkers.

The strength of this study is the validation of the optimized protein extraction protocol in human subjects, providing proof-of-concept that DBS is a viable sampling method for proteomic and metabolomic analysis. The study further benefits from the integration of both targeted and untargeted proteomic approaches, offering a comprehensive overview of the proteome. The use of multi-spot antibody assay adds value by enabling the simultaneous quantification of multiple analytes with high sensitivity, while requiring minimal sample volume (≤ 50 μL). This is particularly advantageous in the context of DBS, where sample availability is limited. Additionally, the combination of 2-D gel electrophoresis and LC–MS/MS enhances the untargeted proteomic profiling. Together, these techniques allow for the detection of low-abundance proteins and yield a robust proteomic landscape from small-volume samples. Additionally, the inclusion of NMR provided critical insights into metabolites that are not detectable via proteomic methods.

In conclusion, this study shows that blood sampling using DBS enables self-sampling in large-scale studies, is compatible with four different omics analysis methods, and can be used to compare the proteome, metabolome, and low abundant cytokines/chemokines. These findings also support the use of DBS for targeted and untargeted proteomic and metabolomic analyses. Additionally, this study demonstrates that levels of GlycA, GlycB, and Glyc can be detected from DBS of healthy individuals, representing, to our knowledge, the first report of these measurements in this context. While proteins involved in T2D could be extracted and detected in healthy individuals, clinical relevance remains to be validated in T2D cohorts.

## Materials and methods

### Ethical declaration

This study is part of a randomized trial^27^ and has been approved by The Swedish Ethical Review Authority in Stockholm, Sweden (2024-07,357-02) and conducted in accordance with the Declaration of Helsinki. It was performed in Linköping, Sweden, and blood samples from in total 16 healthy participants were collected between May and June of 2025. Informed consent was obtained from all participants prior to blood sampling and storing of samples in a biobank in Region Östergötland.

### Participants

For the optimization of the extraction method, two healthy volunteers were recruited. For the validation of the protocol 14 healthy individuals were recruited, of which 7 men, aged between 30- and 63 years. Individuals with type 1 or 2 diabetes mellitus and autoimmune diseases were excluded. Participants were asked to fast a minimum of 8 h prior to sampling.

### Sample collection

The participants’ hands were washed and cleaned with alcohol and allowed to air dry. The third digit was then pricked with a lancet, and the first drop formed was wiped away. The finger was gently massaged to allow formation of an adequately sized blood drop, which was then dropped on to the first circle of a 903™ Five Spot Blood Card (Eastern Business Forms, Inc., Greenville, SC, USA) without touching the filter paper. The finger was then massaged again, and the four remaining circles were filled with one blood drop each. The cards were then left to air dry in room temperature for 4 h and subsequently kept in − 20 °C until analysis, based on previous studies^6–8^.

### Optimization of extraction protocol

#### Evaluation of extraction buffers

To examine the optimal extraction buffer that could be used for both proteomic and metabolomic analysis, PBS-Tween (0.05%), 10 mM PBS, 9 M urea buffer, and the antibody diluent Diluent 100 (Meso Scale Discovery, Rockville, MD, USA) were tested. Each of the 5 DBS were cut in half circles and added to an Eppendorf tube containing 400 μL of either PBS-Tween, PBS, urea buffer, or Diluent 100. One empty DBS circle extracted with 400 μL of PBS-Tween was used as negative control. The tubes were vortexed for 5 min at 750 rpm, after which they were briefly centrifuged, and the eluate was transferred to new tubes. Samples were then kept in − 20 °C until analysis.

To evaluate the effectiveness of the buffers, SDS-PAGE was performed. Briefly, 11 μL sample was added to 11 μL cocktail solution (0.5 M Tris–HCl, pH 6.8; 100% glycerol; 10% SDS; 1% bromophenol blue, DTT) and 3 μL resolution buffer (1.5 M Trizma-Base, pH 8.8) and loaded onto a 12% 10-well Mini-Protean TGX gel (Bio-Rad Laboratories, Hercules, CA, USA), and ran for 15 min at 150 V followed by 45 min at 200 V, 60 mA. Precision Plus Protein All Blue Standards (Bio-Rad Laboratories, Hercules, CA, USA) was used as ladder, and pooled human plasma was used as positive control. After electrophoresis, the separated proteins in the gels were fixed in fixing solution (10% methanol, 7% acetic acid) and stained over night with Lumitein UV Protein Gel Stain (Biotium, Fremont, CA, USA). On subsequent day, the gels were scanned, bands were visualized at 532 nm and photographed using a CCD-camera (VersaDoc 4000MP, Bio-Rad Laboratories, Hercules, CA, USA) in combination with the software Quantity One (Bio-Rad Laboratories, Hercules, CA, USA) to digitize the images. Total protein concentration was measured using the 2-D Quant Kit protocol (Cytiva, Marlborough, MA, USA) according to the manufacturer´s protocol.

#### Examination of extraction time using PBS

After initial assessment of extraction buffers, PBS was determined as most effective based on separation. To further optimize extraction, the time of extraction was investigated. One DBS was added to 400 μL PBS, and the tube was incubated for 5 min with shaking (750 rpm), followed by a brief centrifugation and transfer of the eluate to a new tube. Fresh PBS (400 μL) was then added to the tube containing the DBS, followed by 10 min of shaking, centrifugation, and collection of all eluates. The steps were then repeated with shaking for 20 and 30 min. The eluate samples were analyzed by SDS-PAGE and concentration was measured as described above.

#### 2-D gel electrophoresis

One DBS extract (55 µL) was diluted in 445 µL of 25 mM ammonium bicarbonate (pH 7.4) and desalted using 3 kDa Amicon Ultra centrifugal filters (Merck Millipore, Burlington, MA, USA) at 15,000×*g* for 10 min. The buffer exchange was repeated three times, with the final spin extended to 20 min. The retentate was then recovered by inversion spin for 2 min at 2,000×*g* and dried in a SpeedVac.

Dried sample was reconstituted in 200 µL sample solution (8 M urea, 65 mM DTT, 2% Pharmalyte, 4% CHAPS, bromophenol blue) according to Görg et al.^78^, incubated 60 min in RT (vortexed every 15 min), and centrifuged at 10,000×*g* for 20 min. Protein concentration was measured as previously described, and sample (285 µg) was diluted to 450 µL in rehydration solution (8 M urea, 2% CHAPS, 19 mM DTT, 10 µL IPG buffer pH 3–10) and applied to the IPG strip (pH 3–10). Isoelectric focusing was then performed using an IPGphore system as described previously by Olausson, et al.^79^ and according to the protocol seen in Supplementary Table S11.

Following isoelectric focusing, the IPG strip was equilibrated first in buffer containing 2% DTT for 15 min, and then in buffer containing 2.5% iodoacetamide for 15 min. Finally, the IPG strip was transferred to a 10–15% gradient gel (Serva Electrophoresis GmbH, Heidelberg, Germany) and ran on a BlueTower system (Serva Electrophoresis GmbH, Heidelberg, Germany) according to the protocol seen in Supplementary Table S12.

The gel was fixed overnight in fixing buffer (10% methanol, 7% acetic acid), washed, and stained with Lumitein UV Protein Gel Stain (Biotium Fremont, CA, USA) for 4 h. After washing, gels were imaged at 532 nm using a VersaDoc 4000MP (Bio-Rad Laboratories, Hercules, CA, USA) and digitized using the software Quantity One (Bio-Rad Laboratories, Hercules, CA, USA).

#### Multi-spot assay proinflammatory panel 1 and vascular injury panel 2

To examine whether one drop of blood is enough to detect the desired proinflammatory cytokines (IL-1β, IL-4, IL-6, IL-8, IL-10, and TNF) and CRP, extracted DBS were analyzed on a V-PLEX Proinflammatory Panel 1 and a V-PLEX Vascular Injury Panel 2 (Meso Scale Discovery, Rockville, MD, USA), respectively. The DBS were extracted in PBS for 5, 10, 20, and 30 min and analyzed with no dilution and twofold dilution for the cytokines, and with no dilution and 500- and 1000-fold dilution for CRP. For cytokines, 25 μL of samples were loaded onto a 96-well plate precoated with the capture antibodies human anti-IL-1β, anti-IL-4, anti-IL-6, anti-IL-8, anti-IL-10, and anti-TNF. SULFO-TAG conjugated antibodies anti-hu IL-1β, anti-hu IL-4, anti-hu IL-6, anti-hu IL-8, anti-hu IL-10, and anti-hu TNF were used as detection antibodies. For CRP, 50 μL of samples were loaded onto a 96-well plate precoated with the capture antibody human anti-CRP. SULFO-TAG conjugated antibody anti-hu CRP was used as detection antibody.

Other than testing different dilutions, the assays were done according to the protocols provided by the manufacturer. Plates were read on a MESO Quickplex SQ120 mm instrument (Meso Scale Diagnostics, LLC, Rockville, MD, USA), and raw data was processed in Discovery Workbench (version 4.0, Meso Scale Discovery, Rockville, MD, USA).

### Validation of methods by application on research subjects

#### Multi-spot assay proinflammatory panel 1 and vascular injury panel 2

For the proinflammatory cytokines, 50 μL of 11 undiluted samples, and 40 μL of one sample diluted with 10 μL 10 mM PBS were analyzed as previously described. For CRP, 25 μL of 11 samples 500-fold diluted, and 40 μL of one sample diluted with 10 μL 10 mM PBS and then 500-fold diluted were analyzed as previously described.

#### Liquid chromatography–mass spectrometry

After targeted proteomics, untargeted proteomics using LC–MS/MS was performed to investigate the whole DBS proteome. Prior to analysis, samples were desalted using 3 kDa Amicon Ultra centrifugal filters (Merck Millipore, Burlington, MA, USA) and concentration was measured as previously described. Samples were then dissolved in 20 μL 8 M urea buffer and incubated in RT for 30 min. Subsequently, proteins were reduced and alkylated by incubation with 250 mM DTT and 750 mM iodoacetamide, respectively. Before addition of trypsin, samples were diluted using 25 mM ammonium bicarbonate buffer to eliminate the urea. Trypsin was then added in a 1:25 ratio based on the measured protein amount and incubated overnight at 37 °C and 150 rpm. Following day, samples were dried using a SpeedVac. Dried samples were kept in − 20 °C until analysis.

Samples were then further desalted using C18 filter tips (Thermo Scientific, Waltham, MA, USA), according to the manufacturers protocol. Subsequently, peptide samples (200 ng) were reconstituted in 0.1% formic acid and subjected to LC–MS/MS analysis using a high-resolution trapped ion mobility timsTOF HT mass spectrometer coupled to a nanoElute 2 LC system via a CaptiveSpray ionization source (Bruker Daltonics, Bremen, Germany). Peptides were loaded onto a reverse-phase C18 analytical column (PepSep XTREME, 25 cm × 150 μm × 1.5 μm, Bruker Daltonics, Bremen, Germany) and separated over a 45-min gradient at a flow rate of 400 nL/min and a column temperature of 50 °C. The mobile phases consisted of solvent A (0.1% formic acid in water) and solvent B (0.1% formic acid in acetonitrile). The gradient was programmed as follows: from 2 to 17% B over 25 min; from 25 to 37% B over 35 min; and from 37 to 95% B over 45 min.

Data acquisition was performed in data-independent acquisition parallel accumulation-serial fragmentation (dia-PASEF) mode. MS1 scans were acquired over a mass range of 100–1700 m/z. Collision energy was linearly interpolated based on ion mobility (1/K_0_), ranging from 20 eV at 0.6 Vs/cm^2^ to 59 eV at 1.6 Vs/cm^2^, and held constant outside this range.

Raw dia-PASEF data was processed using Spectronaut (version 18.0, Biognosys AG, Schlieren, Switzerland) against a custom protein sequence database. Search parameters included trypsin as the proteolytic enzyme with a maximum of one missed cleavage allowed; carbamidomethylation of cystein was set as a fixed modification, while oxidation of methionine was considered a variable modification. Peptide and protein identifications were filtered at a 1% false discovery rate. Protein quantification was performed at the protein level group using the top three peptides per protein.

#### Nuclear magnetic resonance spectroscopy

To examine the metabolome of the extracted DBS, 200 µL from 2 different circles per individual (n = 14) were pooled in a new 1.5 mL Eppendorf tube (final volume 400 µL) and mixed (1:1) with Bruker plasma buffer (Bruker Daltonics, Bremen, Germany). The sample (600 µL) was then transferred to 5 mm NMR tubes and quantitative analysis of metabolites was performed using an NMR spectrometer (Bruker Daltonics, Bremen, Germany) as previously described by Jönsson et al.^80^.

### Bioinformatic analysis

Protein–protein interactions and networks were analyzed using the STRING database (version 12.0). Protein accession numbers were entered in the multiple proteins search engine, and the following settings were applied: Organism was set to Homo Sapiens; minimum required interaction score was set to highest confidence (0.900); and maximum number of interactions to show was set to query protein only. Analysis was then based on KEGG Pathways^31–33^, and “Glycolysis/Gluconeogenesis”, “Metabolic pathways”, and “Cholesterol metabolism” was selected. In the network figure, protein–protein interactions (PPI) are represented by a line, and proteins are represented by a node. Purple nodes indicate proteins related to metabolic pathways, red nodes indicate proteins related to glycolysis/gluconeogenesis, and green nodes indicate proteins related to cholesterol metabolism. The PPI enrichment p-value was reported.

### Statistical analysis

Raw data was filed in Excel and statistical analyses were performed in GraphPad Prism 10. To analyze measures of central tendency and dispersion, descriptive statistical analysis was done and presented as mean ± SD. As this is a descriptive exploratory study no inferential statistics were performed.

## Supplementary Information

Below is the link to the electronic supplementary material.

Supplementary Material 1

Supplementary Material 2

## Data Availability

The data that supports the findings of LC–MS/MS and NMR in this study are included in the supplementary material, and further data is available from the corresponding author, SF, upon reasonable request.
