# Supplementary material for: Dried blood spot sample extraction for metabolomics and proteomics profiling for clinical trials: a descriptive exploratory study
Source: Sci Rep. 2026 Apr 13;16:12196. doi: 10.1038/s41598-026-46874-3 (PMC13076783; doi:10.1038/s41598-026-46874-3)
Supplement: Supplementary file 2 — Supplementary Material 2 [file 41598_2026_46874_MOESM2_ESM.docx]

**Supplementary Information**

# Dried Blood Spot Sample Extraction for Metabolomics and Proteomics Profiling for Clinical Trials – a Descriptive Exploratory Study

Siri Fägerstam* ^a^, Emir Johansson ^a, b^, Peder af Geijerstam ^a, c^, Karin Rådholm** ^a, b, d^, Bijar Ghafouri** ^a^

^a^ Department of Health, Medicine and Caring Sciences, Linköping University, Linköping, Sweden

^b^ Primary Health Care Center Kärna, Region Östergötland, Linköping, Sweden

^c^ Primary Healthcare Center Cityhälsan Centrum, Region Östergötland, Norrköping, Sweden

**Karin Rådholm and Bijar Ghafouri are joint last authors of this work.

*Correspondence:

Siri Fägerstam

Department of Health, Medicine and Caring Sciences

Division of clinical medicine

E-mail: [siri.fagerstam@liu.se](mailto:siri.fagerstam@liu.se)

Tel: +46-13-28 25 51

Contents

[Dried Blood Spot Sample Extraction for Metabolomics and Proteomics Profiling for Clinical Trials 1](#_Toc222989311)

[Uncropped gel image of different extraction buffers 2](#_Toc222989312)

[Total protein concentration of DBSs extracted with different buffers 2](#_Toc222989313)

[Total protein concentration of DBSs extracted at different times 2](#_Toc222989314)

[Cytokine concentration of DBSs at different extraction times and dilutions 2](#_Toc222989315)

[CRP concentration of DBSs at different extraction times and dilutions 3](#_Toc222989316)

[Total protein concentration and diameter of DBSs from research subjects 3](#_Toc222989317)

[Cytokine concentrations of DBSs from research subjects 4](#_Toc222989318)

[CRP concentrations of DBSs from research subjects 5](#_Toc222989319)

[Comparison of descriptive statistics with and without outliers 5](#_Toc222989320)

[Proteins identified and quantified by LC-MS 6](#_Toc222989321)

[Protocol for isoelectric focusing in the 1^st^ dimension 81](#_Toc222989322)

[Protocol for SDS-PAGE in the 2^nd^ dimension 81](#_Toc222989323)

## Uncropped gel image of different extraction buffers


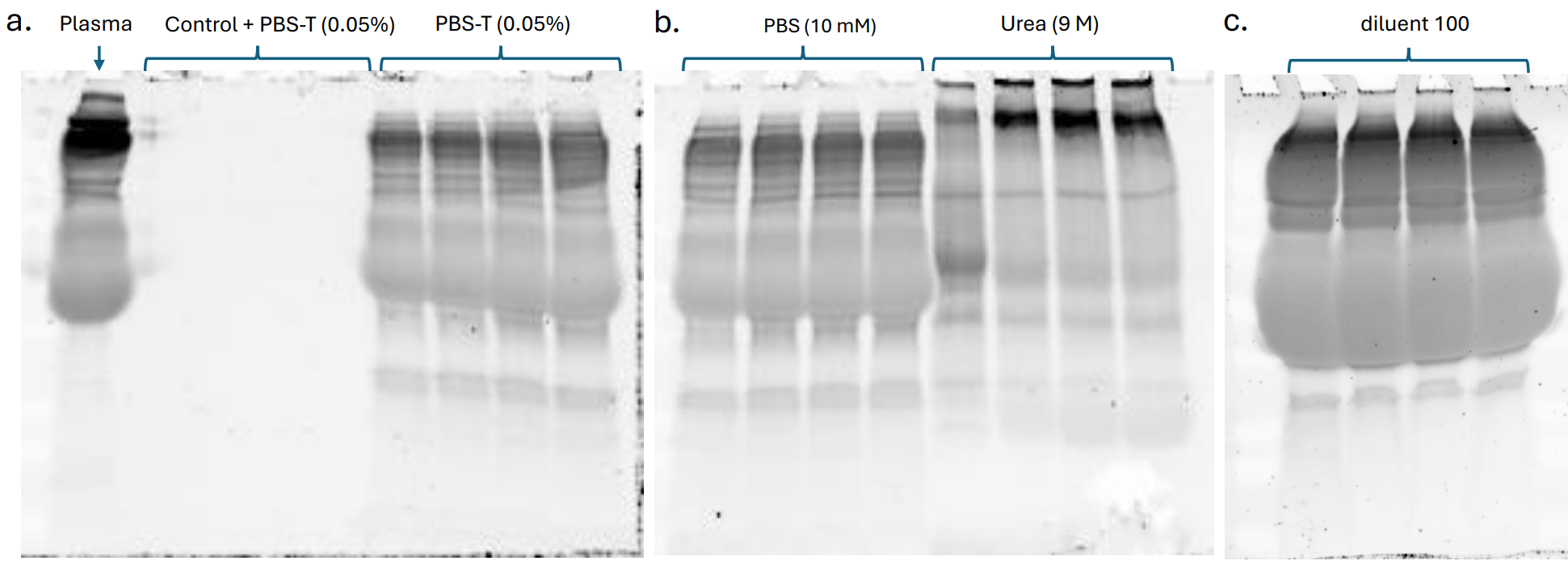


**Supplementary Figure S1:**Uncropped gel images of (a) plasma as positive control, and PBS-T (0.05%) and no DBS as negative control. Extraction of DBSs for 5 minutes with (b) PBS (10 mM), urea (9 M), and (c) diluent 100. For plasma n=1, for negative control and the 4 extraction buffers, n=4.

## Total protein concentration of DBSs extracted with different buffers

**Supplementary Table S1:** Concentration (µg/mL) of DBS with different extraction buffers after 5 minutes.. Measured using the 2-D Quant kit protocol (Cytiva, Marlborough, MA, USA) according to the manufacturers protocol.

| Buffer | 5 min extraction (μg/mL) |
| --- | --- |
| PBS-T | 13.361 |
| PBS | 12.399 |
| Urea | 23.673 |
| Dil. 100 | 37.095 |

## Total protein concentration of DBSs extracted at different times

**Supplementary Table S2:** Concentration (µg/mL) of DBS at different extraction times 5, 10, 20, and 30 minutes, and Diluent 100 without a DBS. Measured using the 2-D Quant kit protocol (Cytiva, Marlborough, MA, USA) according to the manufacturers protocol.

| Time | PBS (μg/mL) | Dil. 100 (μg/mL) |
| --- | --- | --- |
| 5 min | 12.6356667 | – |
| 10 min | 4.87233333 | – |
| 20 min | 1.2965 | – |
| 30 min | 0.5395 | – |
| – | – | 24.4945 |

## Cytokine concentration of DBSs at different extraction times and dilutions

**Supplementary Table S3:** Concentrations (ρg/mL) of proinflammatory cytokines extracted with PBS for 5, 10, 20, and 30 minutes at no dilution and 2-fold dilution. Analyzed on a V-PLEX Proinflammatory Panel 1 (Meso Scale Discovery (MSD), Rockville, MD, USA) according to the manufacturers protocol.

| Sample | IL-10 (ρg/mL) | IL-1β (ρg/mL) | IL-4 (ρg/mL) | IL-6 (ρg/mL) | IL-8 (ρg/mL) | TNF (ρg/mL) |
| --- | --- | --- | --- | --- | --- | --- |
| 5 1:1 | 0.06457903 | 0.90272078 | 0.02066476 | NaN | 3.22601339 | 0.32729398 |
| 10 1:1 | 0.01437087 | 0.24355717 | NaN | NaN | 1.27790482 | 0.06062629 |
| 20 1:1 | NaN | NaN | 0.00500164 | NaN | 0.94453442 | NaN |
| 30 1:1 | NaN | NaN | 0.00328477 | NaN | 0.64914604 | 0.04363267 |
| 5 1:2 | 0.02072381 | 0.83062372 | 0.03546146 | NaN | 3.156347 | 0.05287044 |
| 10 1:2 | NaN | 0.28879856 | 0.0090225 | NaN | 1.27781608 | 0.06856752 |
| 20 1:2 | 0.00016731 | 0.11226084 | 0.00853203 | 0.0480071 | 0.96399322 | NaN |
| 30 1:2 | NaN | 0.08013385 | 0.0050969 | 0.00191212 | 0.80805561 | NaN |

## CRP concentration of DBSs at different extraction times and dilutions

**Supplementary Table S4:** Concentrations (ρg/mL) of CRP extracted with PBS for 5, 10, 20, and 30 minutes at no dilution, 500-fold and 1000-fold dilution. Analyzed on a V-PLEX Vascular Injury 2 (Meso Scale Discovery (MSD), Rockville, MD, USA) according to the manufacturers protocol.

| Sample | CRP (pg/mL) |
| --- | --- |
| 10 1:1 | 8406.487238 |
| 10 1:1000 | 24988.66146 |
| 10 1:500 | 20964.03568 |
| 20 1:1 | 1510.236847 |
| 20 1:1000 | 6073.354317 |
| 20 1:500 | 6701.362545 |
| 30 1:1 | 675.1498177 |
| 30 1:1000 | NaN |
| 30 1:500 | 3653.822993 |
| 5 1:1 | 20068.61437 |
| 5 1:1000 | 75073.13795 |
| 5 1:500 | 54083.65138 |
| P 1:1000 | 1758108.19 |

## Total protein concentration and diameter of DBSs from research subjects

**Supplementary Table S5:** Concentrations (µg/mL) and diameter (mm) of DBSs from research subjects extracted with PBS for 5 minutes. Measured using the 2-D Quant kit protocol (Cytiva, Marlborough, MA, USA) according to the manufacturers protocol.

| Sample | Concentration (μg/mL) | Size (mm) |
| --- | --- | --- |
| 56a | 4.99375 | 7 |
| 56b | 13.17025 | 12 |
| 56c | 5.03525 | 7 |
| 56d | 5.12375 | 7 |
| 56e | 9.92225 | 10 |
| 57a | 7.48725 | 10 |
| 57b | 4.733 | 9 |
| 57c | 5.32925 | 8 |
| 57d | 6.2695 | 8 |
| 57e | 4.50275 | 8 |
| 58a | 13.41975 | 12 |
| 58b | 13.46425 | 12 |
| 58c | 13.2535 | 12 |
| 58d | 9.70325 | 12 |
| 58e | 13.59175 | 12 |
| 59a | 15.88025 | 12 |
| 59b | 15.5 | 12 |
| 59c | 12.56825 | 12 |
| 59d | 12.086 | 12 |
| 59e | 12.355 | 12 |
| 60a | 14.34075 | 12 |
| 60b | 11.99425 | 10 |
| 60c | 12.59625 | 12 |
| 60d | 15.442 | 12 |
| 60e | 17.7135 | 12 |
| 61a | 15.38625 | 12 |
| 61b | 14.22425 | 12 |
| 61c | 7.3625 | 12 |
| 61d | 11.5005 | 12 |
| 61e | 5.981 | 6 |
| 62a | 10.2885 | 7 |
| 62b | 8.58 | 8 |
| 62c | 4.71075 | 6 |
| 62d | 9.35925 | 9 |
| 62e | 11.71675 | 10 |
| 63a | 8.25475 | 9 |
| 63b | 8.81925 | 10 |
| 63c | 3.016 | 6 |
| 63d | 3.36725 | 6 |
| 63e | 4.45875 | 7 |
| 64a | 18.08625 | 12 |
| 64b | 12.20175 | 12 |
| 64c | 6.40125 | 7 |
| 64d | 1.92725 | 5 |
| 64e | 0.23075 | 3 |
| 65a | 12.82075 | 12 |
| 65b | 7.3335 | 9 |
| 65c | 10.0675 | 10 |
| 65d | 10.37825 | 11 |
| 65e | 7.979 | 9 |
| 66a | 15.59525 | 12 |
| 66b | 14.3255 | 12 |
| 66c | 16.47075 | 12 |
| 66d | 16.4815 | 12 |
| 66e | 15.79525 | 12 |
| 67a | 14.08775 | 12 |
| 67b | 11.0535 | 10 |
| 67c | 13.6525 | 12 |
| 67d | 12.4965 | 12 |
| 67e | 7.57925 | 9 |
| 68a | 21.80125 | 12 |
| 68b | 10.073 | 9 |
| 68c | 13.98775 | 12 |
| 68d | 12.926 | 10 |
| 68e | 12.691 | 10 |
| 69a | 19.5615 | 12 |
| 69b | 19.348 | 12 |
| 69c | 19.60725 | 12 |
| 69d | 22.51425 | 12 |
| 69e | 19.0535 | 12 |

## Cytokine concentrations of DBSs from research subjects

**Supplementary Table S6:** Concentrations (ρg/mL) of proinflammatory cytokines of DBSs from research subjects extracted with PBS for 5 minutes. Analyzed on a V-PLEX Proinflammatory Panel 1 (Meso Scale Discovery (MSD), Rockville, MD, USA) according to the manufacturers protocol.

| Sample | IL-10 (ρg/mL) | IL-1B (ρg/mL) | IL-4 (ρg/mL) | IL-6 (ρg/mL) | IL-8 (ρg/mL) | TNF (ρg/mL) |
| --- | --- | --- | --- | --- | --- | --- |
| 56b | 0.03135724 | 10.1010964 | 0.01044355 | 0.14433685 | 4.14700288 | 0.23628312 |
| 66b | NaN | 1,.48270183 | 0.01656786 | 0.02139542 | 2.90692629 | 0.16943178 |
| 58a | 0.01911418 | 0.52652681 | 0.01312283 | 0.06177274 | 2.64226095 | 0.25272668 |
| 67a | 0.04555763 | 0.83202729 | 0.01014692 | 0.17214715 | 3.11559775 | 0.25272668 |
| 59a | 0.03272569 | 2.4634094 | 0.01029521 | 0.89199228 | 4.38994258 | 0.19052413 |
| 68c | 0.02499106 | 7.3194971 | 0.01446827 | 0.03111749 | 3.04555624 | 0.15772231 |
| 60a | 0.02227401 | 0.26046207 | 0.00999869 | 0.03305134 | 1.80110169 | 0.12263587 |
| 69a | 0.03638142 | 1.05861576 | 0.01103748 | 0.05033905 | 4.23319851 | 0.26447712 |
| 61a | 0.02499106 | 4.18099869 | 0.02078803 | 0.065571 | 12.2504013 | 0.20224993 |
| 62e | 0.01731403 | 0.44305922 | 0.01521723 | 0.08636574 | 3.39674487 | 0.19521381 |
| 64b | 0.02227401 | 1.74809391 | 0.00925848 | 0.07882126 | 2.17935494 | 0.20224993 |
| 65a | NaN | 2.34400647 | 0.01297356 | 0.09389281 | 2.7514606 | 0.29034187 |

## CRP concentrations of DBSs from research subjects

**Supplementary Table S7:** Concentration of CRP extracted with PBS for 5 minutes at 500-fold dilution from research subjects. Analyzed on a V-PLEX Proinflammatory Panel 1 (Meso Scale Discovery (MSD), Rockville, MD, USA) according to the manufacturers protocol.

| Sample | CRP (pg/mL) |
| --- | --- |
| 56b | 41433.77642 |
| 58a | 19174.,3526 |
| 59a | 3943.12,23 |
| 60a | 52220.02178 |
| 61a | 76882.48645 |
| 62e | 12344.92513 |
| 64b | 18841.2981 |
| 65a | 40909.6439 |
| 66b | 65087.53167 |
| 67a | 90959.02888 |
| 68c | 26912.74847 |
| 69a | 61939.01344 |

## Comparison of descriptive statistics with and without outliers

**Supplementary Table S8:** Statistical comparison of mean, standard deviation, coefficient of variation, number of missing values, and number of outliers from the results of the multiplex immunoassay analysis of cytokines IL-4, IL-6, IL-8, IL-10, IL-1β, TNF, and CRP. n=14.

| **Outliers excluded** | **Mean** | **SD** | **CV** | **No. of missing values** | **No. of outliers** |
| --- | --- | --- | --- | --- | --- |
| IL-4 | 0.05658 | 0.01507 | 26.64% | 2 | 0 |
| IL-6 | 0.3355 | 0.2071 | 61.73% | 2 | 1 |
| IL-8 | 13.84 | 3.685 | 26.62% | 2 | 1 |
| IL-10 | 0.1219 | 0.03847 | 31.57% | 4 | 0 |
| IL-1β | 6.75 | 5.313 | 78.71% | 2 | 2 |
| TNF | 0.9301 | 0.2158 | 23.21% | 2 | 0 |
| CRP | 0.3395 | 0.264 | 77.77% | 2 | 1 |

| **Outliers included** | **Mean** | **SD** | **CV** | **No. of missing values** | **No. of outliers** |
| --- | --- | --- | --- | --- | --- |
| IL-6 | 0.6346 | 1.055 | 166.2% | 2 | 1 |
| IL-8 | 17.18 | 12.09 | 70.34% | 2 | 1 |
| IL-1β | 12.01 | 13.45 | 112.0% | 2 | 2 |
| CRP | 1.757 | 4.917 | 279.8% | 2 | 1 |

## Proteins identified and quantified by LC-MS

**Supplementary Table S9:** Identification and quantification of proteins from LC-MS. Accession numbers retrieved from the UniProt protein database. Protein name, gene, mean intensity, standard deviation and coefficient of variation are presented. n=5. Comma indicates decimal.

| Protein | Gene | Mean intensity (n=5) | SD | CV (%) |
| --- | --- | --- | --- | --- |
| Hemoglobin subunit beta | HBB | 195650,338 | 5094,094752 | 0,26036729 |
| Complement factor H | CFH | 1069,57205 | 30,9503002 | 0,28937088 |
| UV excision repair protein RAD23 homolog A | RAD23A | 288,129462 | 8,455254475 | 0,29345331 |
| D-dopachrome decarboxylase | DDT | 1206,62727 | 41,21245628 | 0,34155084 |
| Nucleoside diphosphate kinase | NME1-NME2 | 1176,98767 | 41,09956094 | 0,34919279 |
| T-complex protein 1 subunit epsilon;Chaperonin containing TCP1 subunit 5;T-complex protein 1 subunit epsilon;T-complex protein 1 subunit epsilon | CCT5 | 433,252472 | 15,405296 | 0,35557318 |
| Bisphosphoglycerate mutase | BPGM | 2604,95254 | 95,32425185 | 0,3659347 |
| Glucose-6-phosphate 1-dehydrogenase | G6PD | 711,651965 | 26,35176366 | 0,37029004 |
| GTP-binding nuclear protein Ran | RAN | 3582,3355 | 143,4835252 | 0,40053067 |
| ATPase GET3 | GET3 | 267,242868 | 11,14425057 | 0,41700834 |
| F-actin-capping protein subunit alpha-1 | CAPZA1 | 371,728888 | 15,84164157 | 0,42616116 |
| Peroxiredoxin-2 | PRDX2 | 37701,2023 | 1646,99155 | 0,43685385 |
| T-complex protein 1 subunit delta | CCT4 | 233,814224 | 10,32986753 | 0,44179808 |
| 6-phosphogluconate dehydrogenase, decarboxylating | PGD | 1044,80304 | 46,25326117 | 0,44269838 |
| Glutamate--cysteine ligase catalytic subunit | GCLC | 641,823914 | 28,82262294 | 0,44907368 |
| Glucosamine-6-phosphate deaminase 1 | GNPDA1 | 106,29137 | 4,788582931 | 0,45051474 |
| Hypoxanthine-guanine phosphoribosyltransferase | HPRT1 | 357,522675 | 16,30726628 | 0,45611838 |
| UTP--glucose-1-phosphate uridylyltransferase | UGP2 | 152,755835 | 7,003873985 | 0,45850124 |
| 26S proteasome non-ATPase regulatory subunit 3 | PSMD3 | 75,1437088 | 3,48665828 | 0,46399869 |
| Purine nucleoside phosphorylase | PNP | 2912,75898 | 135,2223441 | 0,46424145 |
| 6-phosphogluconolactonase | PGLS | 430,797198 | 20,23543595 | 0,4697207 |
| ITIH4 protein;Inter-alpha-trypsin inhibitor heavy chain H4 | ITIH4 | 2370,20366 | 113,7575924 | 0,4799486 |
| Ubiquitin-like modifier-activating enzyme 1 | UBA1 | 656,666492 | 31,5487576 | 0,48043806 |
| Ras-related protein Rab-7a | RAB7A | 93,3581512 | 4,507612249 | 0,48283007 |
| Acyl-protein thioesterase 1;Acyl-protein thioesterase 1 (Fragment);Acyl-protein thioesterase 1 | LYPLA1 | 81,5738647 | 4,091891147 | 0,50161791 |
| Peptidyl-prolyl cis-trans isomerase FKBP1A | FKBP1A | 236,94725 | 11,91132215 | 0,50269932 |
| Protein phosphatase 2 scaffold subunit Aalpha;Protein phosphatase 2 scaffold subunit Aalpha;Protein phosphatase 2 scaffold subunit Aalpha;Serine/threonine-protein phosphatase 2A 65 kDa regulatory subunit A alpha isoform | PPP2R1A | 500,966577 | 26,35616169 | 0,52610619 |
| Ubiquitin carboxyl-terminal hydrolase 5 | USP5 | 427,87981 | 22,63866681 | 0,52908939 |
| Adenosylhomocysteinase | AHCY | 1305,04702 | 70,14484854 | 0,53748905 |
| Fructose-bisphosphate aldolase A | ALDOA | 2044,2312 | 110,3358814 | 0,53974267 |
| Glutaredoxin-3 | GLRX3 | 115,544589 | 6,239723713 | 0,54002734 |
| Adenylosuccinate lyase | ADSL | 807,64856 | 43,67814093 | 0,54080628 |
| Ras-related protein Rab-1B | RAB1B | 410,187067 | 22,46530029 | 0,54768427 |
| S-formylglutathione hydrolase | ESD | 920,628394 | 51,40872069 | 0,55840903 |
| Syntaxin-7 | STX7 | 21,4182484 | 1,197475517 | 0,55909125 |
| Glycerol-3-phosphate phosphatase | PGP | 95,6125671 | 5,364341213 | 0,56104981 |
| E3 ubiquitin-protein ligase RNF123 | RNF123 | 149,022226 | 8,375637959 | 0,56203951 |
| Thioredoxin | TXN | 559,018335 | 31,57329654 | 0,56479894 |
| Heterogeneous nuclear ribonucleoprotein K;Heterogeneous nuclear ribonucleoprotein K;Heterogeneous nuclear ribonucleoprotein K;Heterogeneous nuclear ribonucleoprotein K;Heterogeneous nuclear ribonucleoprotein K (Fragment);Heterogeneous nuclear ribonucleoprotein K;Heterogeneous nuclear ribonucleoprotein K;Heterogeneous nuclear ribonucleoprotein K | HNRNPK | 47,7478264 | 2,702399554 | 0,56597331 |
| Thioredoxin domain-containing protein 17 | TXNDC17 | 296,434351 | 16,83481394 | 0,56791036 |
| Tubulin-specific chaperone cofactor E-like protein | TBCEL | 129,82924 | 7,439054277 | 0,57298759 |
| Myotrophin | MTPN | 429,512262 | 25,0599761 | 0,58345194 |
| Centrosomal protein 128 (Fragment);Centrosomal protein 128 (Fragment);Centrosomal protein of 128 kDa | CEP128 | 505,974243 | 29,61224906 | 0,5852521 |
| Uncharacterized protein (Fragment);Plasma kallikrein | KLKB1 | 352,749072 | 20,66720019 | 0,58588957 |
| Band 3 anion transport protein | SLC4A1 | 1323,96443 | 77,76060084 | 0,5873315 |
| Cofilin 1;Cofilin 1;Cofilin-1 | CFL1 | 349,794232 | 20,64404493 | 0,59017683 |
| Adenylyl cyclase-associated protein 1 | CAP1 | 96,1301315 | 5,681510898 | 0,5910229 |
| Insulin degrading enzyme;Insulin degrading enzyme;Insulin degrading enzyme;Insulin degrading enzyme;Insulin degrading enzyme;Insulin degrading enzyme;Insulin degrading enzyme;Insulin-degrading enzyme | IDE | 148,721259 | 8,835053728 | 0,59406798 |
| Phosphatidylethanolamine-binding protein 1 | PEBP1 | 1254,19783 | 75,10825148 | 0,5988549 |
| Complement C3 | C3 | 7249,921 | 437,7171951 | 0,60375443 |
| Hemoglobin subunit delta | HBD | 98760,0172 | 5985,099984 | 0,6060246 |
| Stress-induced-phosphoprotein 1 | STIP1 | 824,234192 | 50,18416981 | 0,60885814 |
| Vitamin D-binding protein | GC | 1266,4031 | 77,83038316 | 0,61457827 |
| Alpha-2-antiplasmin | SERPINF2 | 917,07406 | 56,67135762 | 0,61795835 |
| Cullin-associated NEDD8-dissociated protein 1 | CAND1 | 966,634131 | 60,34115939 | 0,6242399 |
| Serine/threonine-protein phosphatase;Serine/threonine-protein phosphatase;Serine/threonine-protein phosphatase;Serine/threonine-protein phosphatase 2A catalytic subunit alpha isoform | PPP2CA | 406,630866 | 25,57049576 | 0,62883804 |
| Ribose-5-phosphate isomerase | RPIA | 452,114648 | 28,86771279 | 0,63850426 |
| Transcriptional activator protein Pur-alpha | PURA | 286,828967 | 18,60993151 | 0,64881632 |
| Transitional endoplasmic reticulum ATPase | VCP | 1114,26849 | 72,39440392 | 0,64970341 |
| NSFL1 cofactor p47 | NSFL1C | 387,838184 | 25,23475341 | 0,6506516 |
| Ribonuclease inhibitor | RNH1 | 1043,46464 | 67,92740733 | 0,65097948 |
| Phosphoribosyl pyrophosphate synthetase 1;Ribose-phosphate pyrophosphokinase 1 | PRPS1 | 472,313794 | 31,18541321 | 0,66026895 |
| L-lactate dehydrogenase A chain | LDHA | 636,447925 | 42,08897818 | 0,66131064 |
| 26S proteasome regulatory subunit 7 | PSMC2 | 126,716104 | 8,441161328 | 0,66614748 |
| Proteasome 26S subunit, ATPase 3 (Fragment);Proteasome 26S subunit, ATPase 3;26S proteasome regulatory subunit 6A;Proteasome 26S subunit, ATPase 3 | PSMC3 | 189,670676 | 12,66917763 | 0,66795658 |
| Biliverdin reductase A | BLVRA | 1087,52537 | 73,10022037 | 0,67217025 |
| Large ribosomal subunit protein P1 | RPLP1 | 149,388763 | 10,09443589 | 0,67571588 |
| Immunoglobulin lambda constant 2;Immunoglobulin lambda constant 3 | IGLC2;IGLC3 | 29446,6664 | 1993,12835 | 0,67686044 |
| Programmed cell death 6-interacting protein | PDCD6IP | 124,157359 | 8,488079934 | 0,683655 |
| Olfactory receptor 10G8 | OR10G8 | 4114,05454 | 284,103996 | 0,69056935 |
| Tubulin beta chain | TUBB | 92,9600754 | 6,435567396 | 0,69229369 |
| Carbonic anhydrase 1 | CA1 | 38999,3531 | 2736,918079 | 0,70178551 |
| L-lactate dehydrogenase;L-lactate dehydrogenase B chain | LDHB | 2036,42629 | 143,0492211 | 0,70245224 |
| Attractin | ATRN | 72,4819214 | 5,110341232 | 0,70505046 |
| Carbonyl reductase [NADPH] 1 | CBR1 | 223,463422 | 15,76693652 | 0,70557125 |
| Eukaryotic peptide chain release factor subunit 1;Eukaryotic peptide chain release factor subunit 1;Eukaryotic peptide chain release factor subunit 1 (Fragment);Eukaryotic peptide chain release factor subunit 1 (Fragment);Eukaryotic peptide chain release factor subunit 1 | ETF1 | 58,9354263 | 4,186812237 | 0,71040671 |
| DNA damage inducible 1 homolog 2;DNA damage inducible 1 homolog 2;Protein DDI1 homolog 2 | DDI2 | 621,016455 | 44,25997944 | 0,7127022 |
| ATP-citrate synthase | ACLY | 348,90769 | 25,01713694 | 0,71701306 |
| Albumin | ALB | 222763,778 | 16029,17283 | 0,71955921 |
| Chloride intracellular channel protein 1 | CLIC1 | 133,737059 | 9,653702139 | 0,72184196 |
| Protein phosphatase methylesterase 1 | PPME1 | 222,277771 | 16,05271067 | 0,72219145 |
| F-actin-capping protein subunit alpha-2 | CAPZA2 | 134,653371 | 9,724670676 | 0,72220031 |
| Inter-alpha-trypsin inhibitor heavy chain H2;Inter-alpha-trypsin inhibitor heavy chain 2 | ITIH2 | 2552,14497 | 184,4723331 | 0,72281291 |
| 5-hydroxymethyl-dUMP N-hydrolase (Fragment);5-hydroxymethyl-dUMP N-hydrolase | DNPH1 | 885,101172 | 64,26781074 | 0,72610694 |
| Actin-related protein 2/3 complex subunit 5 | ARPC5 | 143,174963 | 10,41362475 | 0,7273356 |
| Peroxiredoxin-6 | PRDX6 | 5582,0583 | 410,528926 | 0,73544364 |
| Nicotinate phosphoribosyltransferase | NAPRT | 510,854816 | 37,61664515 | 0,73634708 |
| phosphopyruvate hydratase;Alpha-enolase | ENO1 | 1609,76541 | 118,7665593 | 0,73778799 |
| Bifunctional phosphoribosylaminoimidazole carboxylase/phosphoribosylaminoimidazole succinocarboxamide synthetase (Fragment);Bifunctional phosphoribosylaminoimidazole carboxylase/phosphoribosylaminoimidazole succinocarboxamide synthetase | PAICS | 538,032477 | 39,77313327 | 0,73923295 |
| Importin-9 | IPO9 | 110,608441 | 8,182777335 | 0,73979682 |
| BolA family member 2B;BolA-like protein 2 | BOLA2B;BOLA2 | 120,117671 | 8,911869808 | 0,74192829 |
| Peroxiredoxin-1 | PRDX1 | 36023,7328 | 2694,363906 | 0,74794134 |
| Lactoylglutathione lyase | GLO1 | 99,3611588 | 7,459718413 | 0,75076806 |
| Acylamino-acid-releasing enzyme | APEH | 1168,52366 | 87,74533162 | 0,75090762 |
| DCN1-like protein (Fragment);DCN1-like protein (Fragment);Defective in cullin neddylation 1 domain containing 1 (Fragment);DCN1-like protein;DCN1-like protein 1 | DCUN1D1 | 76,9345001 | 5,796671959 | 0,75345547 |
| GMP reductase 1 | GMPR | 337,107617 | 25,49528022 | 0,75629499 |
| Low molecular weight phosphotyrosine protein phosphatase | ACP1 | 1119,14812 | 84,8068744 | 0,75778061 |
| Leukocyte elastase inhibitor | SERPINB1 | 201,264026 | 15,31318085 | 0,76085037 |
| Alpha-mannosidase 2C1 | MAN2C1 | 29,6764637 | 2,26344308 | 0,76270647 |
| complement subcomponent C1r;complement subcomponent C1r;Complement C1r subcomponent | C1R | 202,219174 | 15,45825123 | 0,76443054 |
| Aldehyde dehydrogenase 1A1 | ALDH1A1 | 2479,08022 | 189,9216813 | 0,76609736 |
| Ubiquitin thioesterase OTU1 | YOD1 | 197,184979 | 15,28052451 | 0,7749335 |
| Eukaryotic translation initiation factor 6 | EIF6 | 85,3382721 | 6,616354269 | 0,77530914 |
| Glutathione S-transferase omega-1 | GSTO1 | 3514,61738 | 273,2297983 | 0,7774098 |
| Ubiquitin-conjugating enzyme E2 N | UBE2N | 368,179205 | 28,69120337 | 0,77927278 |
| ST13 Hsp70 interacting protein (Fragment);Hsc70-interacting protein;Putative protein FAM10A4 | ST13;ST13;ST13P4 | 1430,41704 | 111,7003212 | 0,78089339 |
| RAN binding protein 1 (Fragment);RAN binding protein 1 (Fragment);RAN binding protein 1;Ran-specific GTPase-activating protein | RANBP1 | 190,09046 | 14,89921696 | 0,78379614 |
| Ankyrin-1 | ANK1 | 341,721191 | 26,98199403 | 0,78959089 |
| Complement C5 | C5 | 642,543274 | 51,17979253 | 0,796519 |
| Glyceraldehyde-3-phosphate dehydrogenase | GAPDH | 6853,18701 | 546,1368225 | 0,79690927 |
| Adenosine kinase | ADK | 130,479967 | 10,41064875 | 0,79787334 |
| Hyaluronan-binding protein 2 | HABP2 | 125,804538 | 10,03765068 | 0,79787668 |
| Inter-alpha-trypsin inhibitor heavy chain H1 | ITIH1 | 2174,24443 | 175,0111485 | 0,80492858 |
| ATP-dependent 6-phosphofructokinase, liver type | PFKL | 204,598636 | 16,59713099 | 0,81120438 |
| Damage specific DNA binding protein 1;DNA damage-binding protein 1;Damage specific DNA binding protein 1;DNA damage-binding protein 1 | DDB1 | 69,5027039 | 5,639684344 | 0,8114338 |
| Phosphoglycerate kinase 1 | PGK1 | 2131,6197 | 173,530194 | 0,8140767 |
| Poly(rC)-binding protein 1 | PCBP1 | 287,523901 | 23,4606507 | 0,8159548 |
| Complement C1r subcomponent-like protein | C1RL | 132,273273 | 10,90576718 | 0,82448759 |
| Transaldolase | TALDO1 | 316,026508 | 26,18645834 | 0,82861588 |
| Heterogeneous nuclear ribonucleoprotein A1-like 3;Heterogeneous nuclear ribonucleoprotein A1;Heterogeneous nuclear ribonucleoprotein A1;Heterogeneous nuclear ribonucleoprotein A1;Heterogeneous nuclear ribonucleoprotein A1;Heterogeneous nuclear ribonucleoprotein A1 (Fragment);Heterogeneous nuclear ribonucleoprotein A1 (Fragment);Heterogeneous nuclear ribonucleoprotein A1 (Fragment);Heterogeneous nuclear ribonucleoprotein A1;Heterogeneous nuclear ribonucleoprotein A1;Heterogeneous nuclear ribonucleoprotein A1-like 2 | HNRNPA1L3;HNRNPA1;HNRNPA1;HNRNPA1;HNRNPA1;HNRNPA1;HNRNPA1;HNRNPA1;HNRNPA1;HNRNPA1;HNRNPA1L2 | 95,053772 | 7,911340477 | 0,83230158 |
| Malate dehydrogenase, cytoplasmic | MDH1 | 2021,20159 | 168,5569464 | 0,83394426 |
| Omega-amidase NIT2 (Fragment);Omega-amidase NIT2 | NIT2 | 28,6973469 | 2,399898088 | 0,83627873 |
| Alpha-1-antitrypsin | SERPINA1 | 26781,2438 | 2260,439253 | 0,84403819 |
| Apolipoprotein A-IV | APOA4 | 3880,65366 | 327,5536894 | 0,84406834 |
| Pyruvate kinase;Pyruvate kinase PKM | PKM | 212,608344 | 17,95210937 | 0,84437464 |
| Myosin-9 | MYH9 | 130,447549 | 11,02274161 | 0,84499415 |
| Proteasome 26S subunit ubiquitin receptor, non-ATPase 2 (Fragment);26S proteasome non-ATPase regulatory subunit 2 | PSMD2 | 265,515649 | 22,68793381 | 0,85448575 |
| Nudix hydrolase 5;ADP-sugar pyrophosphatase | NUDT5 | 274,424463 | 23,52024238 | 0,85707528 |
| Rab GDP dissociation inhibitor beta | GDI2 | 984,103833 | 84,41527354 | 0,85778828 |
| 26S proteasome non-ATPase regulatory subunit 1 | PSMD1 | 102,702379 | 8,812000503 | 0,85801328 |
| CXXC motif containing zinc binding protein | CZIB | 280,689832 | 24,26601087 | 0,86451336 |
| Mercaptopyruvate sulfurtransferase;3-mercaptopyruvate sulfurtransferase | MPST | 567,012585 | 49,02812818 | 0,86467443 |
| Nucleosome assembly protein 1 like 1;Nucleosome assembly protein 1 like 1;Nucleosome assembly protein 1 like 1 (Fragment);Nucleosome assembly protein 1 like 1;Nucleosome assembly protein 1 like 1 (Fragment);Nucleosome assembly protein 1 like 1 (Fragment);Nucleosome assembly protein 1 like 1 (Fragment);Nucleosome assembly protein 1 like 1;Nucleosome assembly protein 1 like 1 (Fragment);Nucleosome assembly protein 1 like 1 (Fragment);Nucleosome assembly protein 1 like 1 (Fragment);Nucleosome assembly protein 1-like 1 | NAP1L1 | 233,106625 | 20,19974137 | 0,86654514 |
| Coagulation factor IX | F9 | 451,619012 | 39,2373746 | 0,86881583 |
| Axin interactor, dorsalization-associated protein | AIDA | 91,6386246 | 7,993506819 | 0,87228577 |
| Carbonic anhydrase 2 | CA2 | 25840,693 | 2259,712444 | 0,87447827 |
| Hepatocyte growth factor activator | HGFAC | 105,452509 | 9,253596965 | 0,87751321 |
| Acylphosphatase;Acylphosphatase-1 | ACYP1 | 242,231186 | 21,32275094 | 0,88026448 |
| Calpain-1 catalytic subunit | CAPN1 | 500,837799 | 44,11263616 | 0,8807769 |
| Histidine-rich glycoprotein | HRG | 2537,07969 | 223,8317832 | 0,88224183 |
| Heat shock cognate 71 kDa protein | HSPA8 | 3059,20166 | 270,3097159 | 0,88359561 |
| Carboxypeptidase B2 | CPB2 | 121,032965 | 10,70457313 | 0,88443451 |
| T-complex protein 1 subunit alpha | TCP1 | 231,742093 | 20,50262299 | 0,88471726 |
| Small ubiquitin like modifier 3;SMT3 suppressor of mif two 3 homolog 3 (Yeast), isoform CRA_d;Small ubiquitin-related modifier 3;Small ubiquitin-related modifier 2;Small ubiquitin-related modifier 4 | SUMO3;SUMO3;SUMO3;SUMO2;SUMO4 | 171,519775 | 15,20761991 | 0,88663945 |
| ADP ribosylation factor 1 (Fragment);ADP-ribosylation factor (Fragment);ADP-ribosylation factor (Fragment);ADP ribosylation factor 1 (Fragment);ADP-ribosylation factor 3;ADP-ribosylation factor 3;ADP-ribosylation factor 1 | ARF1;ARF1;ARF1;ARF1;;ARF3;ARF1 | 268,662122 | 23,85518081 | 0,88792498 |
| Afamin | AFM | 18960,8813 | 1695,849451 | 0,89439379 |
| Spectrin beta chain, erythrocytic | SPTB | 1086,30577 | 97,17644771 | 0,89455888 |
| Ubiquitin carboxyl-terminal hydrolase 15 | USP15 | 58,2463303 | 5,249247821 | 0,9012152 |
| T-complex protein 1 subunit theta | CCT8 | 303,21911 | 27,37918939 | 0,90295065 |
| Proteasome activator complex subunit 1 | PSME1 | 658,35072 | 59,47741079 | 0,90343048 |
| Poly(rC) binding protein 2 (Fragment);Poly(rC) binding protein 2;Poly(rC) binding protein 2 (Fragment);Poly(rC)-binding protein 2 | PCBP2 | 210,891559 | 19,0585157 | 0,90371164 |
| Gelsolin | GSN | 2149,51812 | 194,9139029 | 0,90677953 |
| Glutathione peroxidase;Glutathione peroxidase;Glutathione peroxidase;Glutathione peroxidase;Glutathione peroxidase (Fragment);Phospholipid hydroperoxide glutathione peroxidase GPX4;Glutathione peroxidase | GPX4 | 62,1622696 | 5,688203931 | 0,91505731 |
| Hemoglobin subunit alpha | HBA1 | 121999,863 | 11164,65507 | 0,91513669 |
| Immunoglobulin heavy constant gamma 1 | IGHG1 | 34854,5449 | 3225,210541 | 0,92533429 |
| Sorcin | SRI | 413,913501 | 38,32023925 | 0,92580308 |
| Glyoxalase domain-containing protein 4 | GLOD4 | 735,060596 | 68,37025527 | 0,93013087 |
| Beta-adducin | ADD2 | 97,3108459 | 9,054187359 | 0,93043969 |
| Glyoxylate reductase/hydroxypyruvate reductase | GRHPR | 118,682442 | 11,1006311 | 0,9353221 |
| 26S proteasome non-ATPase regulatory subunit 11 | PSMD11 | 86,0214798 | 8,050755761 | 0,93590064 |
| Glutamate--cysteine ligase regulatory subunit | GCLM | 693,440088 | 65,39797809 | 0,94309486 |
| non-specific serine/threonine protein kinase;Serine/threonine-protein kinase WNK1 | WNK1 | 37,616745 | 3,550634729 | 0,94389739 |
| Rieske Fe-S domain containing;Rieske domain-containing protein | RFESD | 82,7986618 | 7,82190357 | 0,94468961 |
| Protein arginine N-methyltransferase 5 | PRMT5 | 82,5989029 | 7,823814197 | 0,94720558 |
| Complement factor I | ;CFI;CFI;CFI | 901,253027 | 85,67192114 | 0,95058678 |
| Apolipoprotein A-II | APOA2 | 5275,7998 | 501,7248419 | 0,95099295 |
| Hemopexin | HPX | 9658,68389 | 923,9748661 | 0,95662605 |
| 14-3-3 protein epsilon | YWHAE | 321,784387 | 30,8470886 | 0,95862602 |
| Glucose-6-phosphate isomerase | GPI | 280,50015 | 27,04770186 | 0,96426693 |
| Phosphoribosylformylglycinamidine synthase | PFAS | 164,970392 | 16,00007048 | 0,96987528 |
| Obg-like ATPase 1 | OLA1 | 408,191357 | 39,7794947 | 0,97453055 |
| Chronophin | PDXP | 67,9092499 | 6,62031304 | 0,97487648 |
| SEC14 like lipid binding 2;CRAL-TRIO domain-containing protein (Fragment);SEC14-like protein 2 | SEC14L2;;SEC14L2 | 88,5653641 | 8,650808488 | 0,97677106 |
| C-1-tetrahydrofolate synthase, cytoplasmic;C-1-tetrahydrofolate synthase, cytoplasmic;C-1-tetrahydrofolate synthase, cytoplasmic;C-1-tetrahydrofolate synthase, cytoplasmic | MTHFD1 | 105,784549 | 10,36094688 | 0,97943858 |
| Thimet oligopeptidase | THOP1 | 123,649178 | 12,14939907 | 0,98257014 |
| Methylosome protein WDR77 | WDR77 | 46,1930992 | 4,547852757 | 0,98453077 |
| TSC22 domain family protein 3;TSC22 domain family protein 4 | TSC22D4 | 55,2723259 | 5,468320632 | 0,98934151 |
| Proteasome activator complex subunit 2 | PSME2 | 265,987238 | 26,36338974 | 0,99115243 |
| WD repeat domain 1;WD repeat-containing protein 1 | WDR1 | 355,235712 | 35,23355172 | 0,99183586 |
| ubiquitinyl hydrolase 1;Ubiquitin thioesterase OTUB1 | OTUB1 | 148,532449 | 14,75901757 | 0,99365611 |
| G protein pathway suppressor 1;G protein pathway suppressor 1;COP9 signalosome complex subunit 1 | GPS1 | 54,523877 | 5,424422623 | 0,99487104 |
| Heat shock protein HSP 90-beta | HSP90AB1 | 46,1490013 | 4,647439376 | 1,00705091 |
| Complement C4-B | C4B | 3873,98306 | 390,9312757 | 1,00911973 |
| Large ribosomal subunit protein bL21m | MRPL21 | 72,8437447 | 7,401532787 | 1,01608351 |
| Acetyl-CoA acetyltransferase, cytosolic | ACAT2 | 304,877289 | 31,02054476 | 1,0174764 |
| Heat shock 70 kDa protein 4 | HSPA4 | 337,856726 | 34,54398885 | 1,02244491 |
| F-actin-capping protein subunit beta | CAPZB | 246,631073 | 25,25858852 | 1,02414461 |
| Exportin-7 | XPO7 | 85,7341064 | 8,784181944 | 1,0245843 |
| Dematin | DMTN | 113,535046 | 11,63866033 | 1,0251161 |
| Microtubule-associated protein RP/EB family member 1 | MAPRE1 | 119,583636 | 12,26796009 | 1,02588954 |
| ubiquitinyl hydrolase 1;ubiquitinyl hydrolase 1;Ataxin 3;ubiquitinyl hydrolase 1;ubiquitinyl hydrolase 1 (Fragment);ubiquitinyl hydrolase 1;ubiquitinyl hydrolase 1 (Fragment);Ataxin 3 (Fragment);ubiquitinyl hydrolase 1 (Fragment);ubiquitinyl hydrolase 1 (Fragment);Ataxin 3 (Fragment);ubiquitinyl hydrolase 1 (Fragment);ubiquitinyl hydrolase 1;Ataxin-3;ubiquitinyl hydrolase 1 (Fragment) | ATXN3 | 133,92778 | 13,74896961 | 1,0265958 |
| Glutathione synthetase | GSS | 134,40023 | 13,8127206 | 1,02773043 |
| NIF3-like protein 1 | NIF3L1 | 133,552254 | 13,76049794 | 1,03034562 |
| Dual specificity mitogen-activated protein kinase kinase 1;Dual specificity mitogen-activated protein kinase kinase 1;Dual specificity mitogen-activated protein kinase kinase 1;Dual specificity mitogen-activated protein kinase kinase 1;Dual specificity mitogen-activated protein kinase kinase 1;Dual specificity mitogen-activated protein kinase kinase 1;Dual specificity mitogen-activated protein kinase kinase 1;Dual specificity mitogen-activated protein kinase kinase 2;Dual specificity mitogen-activated protein kinase kinase 1 | MAP2K1;MAP2K1;MAP2K1;MAP2K1;MAP2K1;MAP2K1;MAP2K1;MAP2K2;MAP2K1 | 13,3378393 | 1,374266868 | 1,03035195 |
| 26S proteasome non-ATPase regulatory subunit 5 | PSMD5 | 346,357611 | 35,99779949 | 1,03932463 |
| Eukaryotic translation initiation factor 5 | EIF5 | 75,3528778 | 7,853500976 | 1,04222973 |
| Corticosteroid-binding globulin | SERPINA6 | 383,717541 | 40,02667296 | 1,04312857 |
| Alpha-1-antichymotrypsin | SERPINA3 | 2556,90479 | 268,7130337 | 1,05093093 |
| Complement factor B |  | 2510,33843 | 263,899218 | 1,05124956 |
| MAGUK p55 scaffold protein 1 (Fragment);55 kDa erythrocyte membrane protein | MPP1 | 159,721051 | 16,81407746 | 1,05271518 |
| Vesicle-fusing ATPase | NSF | 96,9435181 | 10,22054514 | 1,05427834 |
| Peroxiredoxin-5, mitochondrial | PRDX5 | 107,344864 | 11,31810202 | 1,05436829 |
| Serine/threonine-protein phosphatase 2A activator | PTPA | 808,548193 | 85,48636364 | 1,05728223 |
| Serpin family A member 10;Protein Z-dependent protease inhibitor | SERPINA10 | 266,719015 | 28,28305748 | 1,06040649 |
| Tubulin-specific chaperone A | TBCA | 202,008868 | 21,43553593 | 1,06111856 |
| Protein disulfide-isomerase A6 | PDIA6 | 55,7464996 | 5,915972817 | 1,06122768 |
| Selenoprotein P (Fragment);Selenoprotein P | SELENOP | 307,093085 | 32,73051682 | 1,06581745 |
| Phosphoribosyl pyrophosphate synthase-associated protein 2 | PRPSAP2 | 111,175671 | 11,8864003 | 1,06915481 |
| Glucose 1,6-bisphosphate synthase | PGM2L1 | 208,134067 | 22,40146726 | 1,07629989 |
| protein-tyrosine-phosphatase;protein-tyrosine-phosphatase;Protein tyrosine phosphatase non-receptor type 11;Protein tyrosine phosphatase non-receptor type 11;protein-tyrosine-phosphatase;Protein tyrosine phosphatase non-receptor type 11;Tyrosine-protein phosphatase non-receptor type 11 | PTPN11 | 58,31511 | 6,2836966 | 1,07754176 |
| Dehydrogenase/reductase 11 (Fragment);Dehydrogenase/reductase SDR family member 11 | DHRS11 | 69,147551 | 7,454342141 | 1,07803415 |
| Aminopeptidase;Puromycin-sensitive aminopeptidase | NPEPPS | 97,6593018 | 10,56164312 | 1,08147846 |
| KRAS proto-oncogene, GTPase;KRAS proto-oncogene, GTPase | KRAS | 87,7577683 | 9,532375061 | 1,08621439 |
| 26S proteasome non-ATPase regulatory subunit 9 | PSMD9 | 987,926917 | 107,6551525 | 1,08970766 |
| Phosphatidylinositol 5-phosphate 4-kinase type-2 alpha | PIP4K2A | 205,680161 | 22,54376407 | 1,09605924 |
| Fumarate hydratase, mitochondrial | FH | 152,930057 | 16,84978917 | 1,10179709 |
| Bifunctional coenzyme A synthase | COASY | 104,668195 | 11,53614305 | 1,10216318 |
| Peptidyl-prolyl cis-trans isomerase A | PPIA | 1926,15742 | 212,4185885 | 1,10281011 |
| Ubiquitin-conjugating enzyme E2 K | UBE2K | 105,025479 | 11,58603065 | 1,1031638 |
| Alpha-2-HS-glycoprotein | AHSG | 848,121021 | 94,36550499 | 1,11264198 |
| 4-trimethylaminobutyraldehyde dehydrogenase | ALDH9A1 | 172,32081 | 19,1734305 | 1,11265903 |
| Aspartate--tRNA ligase, cytoplasmic | DARS1 | 91,436763 | 10,20772975 | 1,11637042 |
| Proteasome subunit beta (Fragment);Proteasome subunit alpha type (Fragment);Proteasome subunit alpha type (Fragment);Proteasome subunit alpha type (Fragment);Proteasome 20S subunit alpha 4;Proteasome subunit alpha type-4 | PSMA4 | 260,024954 | 29,03657793 | 1,11668428 |
| Parkinsonism associated deglycase;Parkinson disease protein 7 | PARK7 | 847,892383 | 94,75705483 | 1,11755993 |
| Protein AMBP | AMBP | 2686,59136 | 300,5479568 | 1,11869621 |
| COP9 signalosome subunit 7B;COP9 signalosome complex subunit 7b | COPS7B | 15,872576 | 1,779733224 | 1,121263 |
| Histone-arginine methyltransferase CARM1 | CARM1 | 178,399551 | 20,12272105 | 1,12795805 |
| Alpha-1B-glycoprotein | A1BG | 6531,76162 | 736,9476561 | 1,12825253 |
| BMP-2-inducible protein kinase (Fragment);BMP-2-inducible protein kinase | BMP2K | 15,2925697 | 1,749422434 | 1,14396891 |
| Spectrin alpha chain, erythrocytic 1 | SPTA1 | 343,280774 | 39,27909722 | 1,14422654 |
| Calmodulin-1;Calmodulin-2;Calmodulin-3 | CALM1;CALM2;CALM3 | 329,797717 | 37,74410136 | 1,14446218 |
| Phosphoglycerate mutase 1 | PGAM1 | 261,168188 | 29,95267624 | 1,14687307 |
| Eukaryotic translation initiation factor 4E | EIF4E | 140,636192 | 16,15322111 | 1,14858209 |
| Clusterin | CLU | 2278,74187 | 262,9531509 | 1,15394005 |
| Angiotensinogen | AGT | 1792,09641 | 207,2308232 | 1,15635979 |
| Autophagy related 7 (Fragment);Autophagy related 7 (Fragment);Autophagy related 7 (Fragment);Ubiquitin-like modifier-activating enzyme ATG7 | ATG7 | 128,909639 | 14,91116931 | 1,15671485 |
| GMP reductase;GMP reductase;GMP reductase (Fragment);GMP reductase (Fragment);GMP reductase;GMP reductase;GMP reductase (Fragment);GMP reductase (Fragment);GMP reductase;GMP reductase 2 | GMPR2 | 72,8366531 | 8,488595389 | 1,16542908 |
| Cytokine receptor-like factor 3 | CRLF3 | 55,7173965 | 6,49540987 | 1,16577771 |
| Bleomycin hydrolase (Fragment);Bleomycin hydrolase | BLMH | 136,250053 | 15,91015466 | 1,16771732 |
| Platelet-activating factor acetylhydrolase IB subunit alpha1 (Fragment);Platelet-activating factor acetylhydrolase IB subunit alpha1 | PAFAH1B3 | 422,429193 | 49,38467202 | 1,1690639 |
| 26S proteasome non-ATPase regulatory subunit 13 | PSMD13 | 72,497187 | 8,48615897 | 1,1705501 |
| C-type lectin domain family 3 member B;Tetranectin | CLEC3B | 178,2457 | 20,88171436 | 1,17151294 |
| Prolyl endopeptidase | PREP | 91,1945587 | 10,7126772 | 1,17470575 |
| Pigment epithelium-derived factor | SERPINF1 | 634,619031 | 75,00494928 | 1,18188938 |
| Phosphatidylinositol transfer protein alpha isoform;Phosphatidylinositol transfer protein alpha isoform (Fragment);Phosphatidylinositol transfer protein alpha isoform | PITPNA | 158,086456 | 18,69359176 | 1,18249167 |
| Superoxide dismutase [Cu-Zn] | SOD1 | 2408,67627 | 285,244695 | 1,18423841 |
| Adenylate kinase isoenzyme 1 | AK1 | 1733,99817 | 205,7594299 | 1,18661849 |
| Vitamin K-dependent protein S (Fragment);Vitamin K-dependent protein S;Vitamin K-dependent protein S | PROS1 | 360,361273 | 42,84162458 | 1,18885207 |
| Calcium-regulated heat-stable protein 1 | CARHSP1 | 73,3800354 | 8,762059698 | 1,19406589 |
| Importin subunit beta-1 | KPNB1 | 435,626849 | 52,03545725 | 1,1944961 |
| Tripeptidyl-peptidase 2 (Fragment);Tripeptidyl-peptidase 2;Tripeptidyl-peptidase 2 | TPP2 | 312,950391 | 37,44775649 | 1,1966036 |
| Rho GTPase-activating protein 1 | ARHGAP1 | 121,10789 | 14,5065859 | 1,19782335 |
| Prothrombin | F2 | 1748,32158 | 209,4556283 | 1,19803834 |
| Radixin | RDX | 86,9297928 | 10,42379657 | 1,19910519 |
| Proteasome subunit alpha type;Proteasome subunit alpha type-2 | PSMA2 | 325,045221 | 39,05927631 | 1,20165669 |
| Complement C2 | C2 | 264,236603 | 31,78063377 | 1,20273397 |
| Alpha-1-acid glycoprotein 1 | ORM1 | 4278,18052 | 515,5909166 | 1,205164 |
| Serotransferrin | TF | 27517,9879 | 3320,946456 | 1,2068275 |
| Rab GDP dissociation inhibitor alpha | GDI1 | 122,822603 | 14,82799941 | 1,20726959 |
| Coagulation factor XIII B chain | F13B | 88,3296204 | 10,6763569 | 1,20869498 |
| RAB11A, member RAS oncogene family (Fragment);RAB11A, member RAS oncogene family;Ras-related protein Rab-11A;Ras-related protein Rab-11B | RAB11A;RAB11A;RAB11A;RAB11B | 220,737805 | 27,1643387 | 1,2306156 |
| Uroporphyrinogen decarboxylase | UROD | 280,858606 | 34,70380515 | 1,23563261 |
| adenine phosphoribosyltransferase;Adenine phosphoribosyltransferase | APRT | 329,346979 | 40,83851063 | 1,23998437 |
| Immunoglobulin lambda variable 3-19 | IGLV3-19 | 153,937593 | 19,11764266 | 1,24190864 |
| Chromosome 11 open reading frame 54;Cofactor required for Sp1 transcriptional activation subunit 6;Chromosome 11 open reading frame 54 (Fragment);Ester hydrolase C11orf54 | C11orf54;;C11orf54;C11orf54 | 81,1801773 | 10,12408442 | 1,24711287 |
| Gamma-glutamylcyclotransferase | GGCT | 43,2856926 | 5,429631603 | 1,25437097 |
| 14-3-3 protein zeta/delta | YWHAZ | 1057,90692 | 133,0404987 | 1,25758227 |
| Heme-binding protein 1 | HEBP1 | 340,046307 | 42,85009666 | 1,26012534 |
| Protein-L-isoaspartate O-methyltransferase;Protein-L-isoaspartate(D-aspartate) O-methyltransferase | PCMT1 | 161,900024 | 20,4381165 | 1,2623912 |
| 26S proteasome regulatory subunit 6B | PSMC4 | 149,433594 | 18,8721734 | 1,26291371 |
| Actinin alpha 1;Actinin alpha 1;Actinin alpha 1;Actinin alpha 1;Actinin alpha 1;Actinin alpha 1 (Fragment);Alpha-actinin-1 | ACTN1 | 722,462769 | 91,25949697 | 1,26317232 |
| Proteasome subunit alpha type-7 | PSMA7 | 687,611768 | 87,00934708 | 1,26538479 |
| Proteasome subunit beta type-1 | PSMB1 | 277,644089 | 35,15292132 | 1,26611452 |
| Apolipoprotein A-I | APOA1 | 54291,5719 | 6895,068291 | 1,27000712 |
| Ras homolog family member A;Uncharacterized protein;Transforming protein RhoA (Fragment);Ras homolog family member A;Ras homolog family member C (Fragment);Rho-related GTP-binding protein RhoC;Transforming protein RhoA;Ras homolog family member C;Ras homolog family member C (Fragment);Ras homolog family member C (Fragment) | RHOA;;RHOA;RHOA;RHOC;RHOC;RHOA;RHOC;RHOC;RHOC | 140,559985 | 17,90374011 | 1,27374374 |
| Adapter SH3BGRL | SH3BGRL | 237,889832 | 30,71559721 | 1,29116898 |
| COP9 signalosome complex subunit 4 | COPS4 | 199,00535 | 25,71091185 | 1,29197089 |
| Hydroxyacylglutathione hydrolase, mitochondrial (Fragment);Hydroxyacylglutathione hydrolase, mitochondrial;Hydroxyacylglutathione hydrolase, mitochondrial | HAGH | 234,098633 | 30,32926933 | 1,29557652 |
| 78 kDa glucose-regulated protein;78 kDa glucose-regulated protein;Endoplasmic reticulum chaperone BiP | HSPA5 | 36,9743935 | 4,806043498 | 1,29983025 |
| Alpha-soluble NSF attachment protein | NAPA | 398,87041 | 51,9172495 | 1,30160694 |
| Protein argonaute-2 | AGO2 | 90,3009445 | 11,76775705 | 1,30317098 |
| DnaJ heat shock protein family (Hsp40) member B2 (Fragment);DnaJ heat shock protein family (Hsp40) member B2 (Fragment);DnaJ homolog subfamily B member 2 | DNAJB2 | 22,093877 | 2,886125298 | 1,30630097 |
| HCG2044781;Ubiquitin-conjugating enzyme E2 variant 1 | PEDS1-UBE2V1;UBE2V1 | 1168,04415 | 153,0413812 | 1,31023627 |
| UV excision repair protein RAD23 homolog B | RAD23B | 174,421204 | 22,92832657 | 1,3145378 |
| Methanethiol oxidase | SELENBP1 | 4585,60947 | 604,6816574 | 1,31865058 |
| S-phase kinase-associated protein 1 |  | 64,0617348 | 8,463820671 | 1,32119754 |
| Carboxypeptidase N catalytic chain | CPN1 | 106,986072 | 14,14260922 | 1,32191125 |
| Heat shock protein family A (Hsp70) member 1B;Heat shock 70 kDa protein 1A;Heat shock 70 kDa protein 1B | HSPA1B;HSPA1A;HSPA1B | 278,512811 | 36,87231131 | 1,32390001 |
| Insulin-like growth factor-binding protein complex acid labile subunit | IGFALS | 605,447327 | 80,49290818 | 1,3294783 |
| Arginase-1 | ARG1 | 95,4687347 | 12,71431665 | 1,33177806 |
| CB1 cannabinoid receptor-interacting protein 1 | CNRIP1 | 161,927051 | 21,59607185 | 1,33369142 |
| Ubiquitin-conjugating enzyme E2 L3 | UBE2L3 | 277,381558 | 37,01139088 | 1,33431332 |
| COP9 signalosome subunit 8;COP9 signalosome complex subunit 8 | COPS8 | 106,695793 | 14,24658043 | 1,33525231 |
| Transforming growth factor beta induced (Fragment);Transforming growth factor-beta-induced protein ig-h3 | TGFBI | 189,937952 | 25,41697294 | 1,33817242 |
| Proteasome inhibitor PI31 subunit | PSMF1 | 180,089334 | 24,1269677 | 1,33972219 |
| Immunoglobulin lambda variable 3-25 | IGLV3-25 | 132,598335 | 17,77545085 | 1,34054857 |
| NAD(P)H-hydrate epimerase | NAXE | 97,7310867 | 13,11406446 | 1,3418519 |
| Golgi-associated plant pathogenesis-related protein 1 | GLIPR2 | 113,252098 | 15,22172832 | 1,34405707 |
| Leucine-rich alpha-2-glycoprotein | LRG1 | 400,475964 | 53,93004451 | 1,34664872 |
| Kallistatin | SERPINA4 | 483,470276 | 65,16051073 | 1,34776664 |
| Cytosol aminopeptidase | LAP3 | 74,5993454 | 10,08330876 | 1,35166183 |
| Glia maturation factor beta (Fragment);Glia maturation factor gamma;Glia maturation factor;Glia maturation factor;Glia maturation factor;Glia maturation factor gamma;Glia maturation factor beta | GMFB;GMFG;GMFG;GMFG;GMFG;GMFG;GMFB | 76,2882057 | 10,32915474 | 1,35396483 |
| RNA 3'-terminal phosphate cyclase | RTCA | 180,408472 | 24,43198507 | 1,3542593 |
| Plasminogen | PLG | 2822,69019 | 382,398023 | 1,35472899 |
| Apolipoprotein D (Fragment);Apolipoprotein D | APOD | 688,898535 | 93,32761396 | 1,35473672 |
| Serine/threonine-protein kinase OSR1 | OXSR1 | 85,1383011 | 11,55737151 | 1,35748204 |
| Complement C1s subcomponent | C1S | 245,75177 | 33,54992945 | 1,36519584 |
| Eukaryotic translation initiation factor 3 subunit J | EIF3J | 92,1863876 | 12,5911718 | 1,36583851 |
| C4b-binding protein alpha chain | C4BPA | 1409,94731 | 193,3613494 | 1,37140834 |
| Rac family small GTPase 1;Ras-related C3 botulinum toxin substrate 3;Ras-related C3 botulinum toxin substrate 1 | RAC1;RAC3;RAC1 | 133,891177 | 18,40000391 | 1,37425067 |
| Elongation factor 1-alpha;Elongation factor 1-alpha;Elongation factor 1-alpha;Elongation factor 1-alpha;Elongation factor 1-alpha 1;Putative elongation factor 1-alpha-like 3 | EEF1A1;EEF1A1;EEF1A1;EEF1A1;EEF1A1;EEF1A1P5 | 59,0058678 | 8,108894086 | 1,37425215 |
| Moesin | MSN | 653,208459 | 90,17009474 | 1,38041836 |
| Putative protein-lysine deacylase ABHD14B | ABHD14B | 266,889957 | 36,87996206 | 1,38184151 |
| Proteasome subunit alpha type-3 | PSMA3 | 95,7540115 | 13,24438294 | 1,38316742 |
| Rho GDP dissociation inhibitor beta (Fragment);Rho GDP dissociation inhibitor beta (Fragment);Rho GDP dissociation inhibitor beta (Fragment);Rho GDP dissociation inhibitor beta (Fragment);Rho GDP-dissociation inhibitor 2 | ARHGDIB | 454,473364 | 62,87186041 | 1,38340034 |
| Vimentin | VIM | 167,042236 | 23,13852553 | 1,38519012 |
| Adducin 1;Adducin 1;Adducin 1;Alpha-adducin | ADD1 | 132,887004 | 18,43850156 | 1,38753234 |
| Cleavage and polyadenylation specificity factor subunit 1 | CPSF1 | 1643,72737 | 228,701596 | 1,39135966 |
| Plastin-2 | LCP1 | 453,685431 | 63,13096282 | 1,391514 |
| AP-2 complex subunit alpha-1 | AP2A1 | 104,797133 | 14,60838742 | 1,39396823 |
| Glutathione reductase, mitochondrial | GSR | 127,947612 | 17,85522903 | 1,39551092 |
| Aspartate aminotransferase, cytoplasmic | GOT1 | 225,927307 | 31,53835 | 1,39595122 |
| T-complex protein 1 subunit beta | CCT2 | 251,953799 | 35,306743 | 1,40131814 |
| Immunoglobulin heavy variable 3-72 | IGHV3-72 | 5494,43779 | 770,2310717 | 1,40183782 |
| Ubiquitin carboxyl-terminal hydrolase 14 | USP14 | 198,133093 | 27,78002904 | 1,4020893 |
| Nucleosome assembly protein 1-like 4 | NAP1L4 | 281,987692 | 39,53835255 | 1,40213044 |
| Apolipoprotein F | APOF | 283,532602 | 40,0266894 | 1,41171382 |
| Flavin reductase (NADPH) | BLVRB | 4811,8939 | 679,7860238 | 1,41272031 |
| MBL associated serine protease 1;Mannan-binding lectin serine protease 1 | MASP1 | 115,534962 | 16,32953276 | 1,41338453 |
| Fibrinogen alpha chain | FGA | 49,7955444 | 7,051098325 | 1,41600989 |
| Calpastatin;Calpastatin;Calpastatin;Calpastatin;Calpastatin;Calpastatin;Calpastatin;Calpastatin;Calpastatin;Calpastatin (Fragment);Calpastatin;Calpastatin;Calpastatin;Calpastatin (Fragment);Calpastatin (Fragment);Calpastatin | CAST | 617,024066 | 87,56402778 | 1,41913472 |
| AP-2 complex subunit mu | AP2M1 | 26,1604919 | 3,712769263 | 1,41922762 |
| Proteasome assembly chaperone 3 | PSMG3 | 64,3531265 | 9,161127485 | 1,42357147 |
| Argininosuccinate lyase | ASL | 85,2231491 | 12,20251722 | 1,4318313 |
| Mitogen-activated protein kinase kinase 3 (Fragment);Dual specificity mitogen-activated protein kinase kinase 3 | MAP2K3 | 129,735214 | 18,58226649 | 1,43232249 |
| Trafficking kinesin protein 1;Trafficking kinesin protein 1;Trafficking kinesin protein 1;Trafficking kinesin protein 1;Trafficking kinesin protein 1;Trafficking kinesin protein 1;Trafficking kinesin protein 1 (Fragment);Trafficking kinesin-binding protein 1 | TRAK1 | 1061,96764 | 152,2091874 | 1,43327519 |
| Immunoglobulin heavy variable 3-74 | IGHV3-74 | 788,512537 | 113,4159016 | 1,43835255 |
| Annexin A7 | ANXA7 | 558,7367 | 80,56417488 | 1,44189875 |
| COP9 signalosome complex subunit 6 | COPS6 | 39,9889984 | 5,781530025 | 1,44578015 |
| Proteasome subunit alpha type-5 | PSMA5 | 292,842191 | 42,45360523 | 1,44970932 |
| Transketolase | TKT | 404,995044 | 58,74334767 | 1,45047078 |
| Proteasome 26S subunit, ATPase 5;26S proteasome regulatory subunit 8 | PSMC5 | 199,058496 | 28,92101081 | 1,45289005 |
| Monocyte differentiation antigen CD14 (Fragment);Monocyte differentiation antigen CD14 | CD14 | 75,7212044 | 11,03935801 | 1,4578952 |
| Alpha-centractin;Actin related protein 1A | ACTR1A | 145,497365 | 21,23171897 | 1,4592511 |
| Aldehyde dehydrogenase family 16 member A1 | ALDH16A1 | 162,891978 | 23,79845997 | 1,46099644 |
| Glucosamine-6-phosphate deaminase 2 | GNPDA2 | 49,5644951 | 7,243769127 | 1,46148349 |
| Biotinidase | BTD | 32,9982018 | 4,829060871 | 1,46343152 |
| Lumican | LUM | 502,873224 | 73,63120484 | 1,46421009 |
| Crk-like protein | CRKL | 49,1098839 | 7,191945074 | 1,4644598 |
| Latexin | LXN | 386,008423 | 56,64427013 | 1,46743612 |
| Serine/threonine-protein phosphatase 6 catalytic subunit | PPP6C | 84,0296204 | 12,42639777 | 1,4788116 |
| Ubiquitin carboxyl-terminal hydrolase isozyme L3 | UCHL3 | 311,874976 | 46,26860507 | 1,4835626 |
| Neutrophil gelatinase-associated lipocalin;Lipocalin 2 | LCN2 | 117,197624 | 17,40729513 | 1,4852942 |
| Fascin | FSCN1 | 152,333659 | 22,63991654 | 1,48620579 |
| GDP-L-fucose synthase | GFUS | 573,190924 | 85,94715139 | 1,49945067 |
| protein-ribulosamine 3-kinase (Fragment);Ketosamine-3-kinase | FN3KRP | 61,0529449 | 9,207433108 | 1,5081063 |
| Importin subunit alpha;Importin subunit alpha;Importin subunit alpha-3 | KPNA4 | 86,3419327 | 13,03755553 | 1,50999116 |
| N-acetylneuraminate lyase | NPL | 74,8631454 | 11,30962603 | 1,51070677 |
| RNA exonuclease 2;RNA exonuclease 2 (Fragment);Oligoribonuclease, mitochondrial | REXO2 | 124,174054 | 18,85505848 | 1,51843786 |
| Adenosine 5'-monophosphoramidase HINT1 | HINT1 | 100,694119 | 15,31058597 | 1,52050449 |
| Cyclin-dependent kinase 2;Cyclin-dependent kinase 2;Cyclin-dependent kinase 2;Cyclin-dependent kinase 3 | CDK2;CDK2;CDK2;CDK3 | 106,393207 | 16,21036133 | 1,52362747 |
| Ceruloplasmin | CP | 8205,69766 | 1252,436231 | 1,52630073 |
| Glutathione peroxidase 3 | GPX3 | 334,660339 | 51,13412413 | 1,52794097 |
| Family with sequence similarity 114 member A2;Protein FAM114A2 | FAM114A2 | 121,227153 | 18,67498045 | 1,54049485 |
| LanC-like protein 2 | LANCL2 | 74,771653 | 11,52013124 | 1,54070838 |
| Phosphopantothenoylcysteine decarboxylase | PPCDC | 90,8752411 | 14,00709202 | 1,54135404 |
| Lecithin-cholesterol acyltransferase (Fragment);Lecithin-cholesterol acyltransferase (Fragment);Lecithin-cholesterol acyltransferase (Fragment);Phosphatidylcholine-sterol acyltransferase | LCAT | 51,5207016 | 7,962601427 | 1,54551494 |
| Exopolyphosphatase PRUNE1 | PRUNE1 | 14,6695652 | 2,268662354 | 1,54650961 |
| Protein-lysine N-trimethyltransferase SMYD5 | SMYD5 | 18,5260036 | 2,867659979 | 1,54791073 |
| Ubiquitin-like protein NEDD8 | NEDD8 | 299,775577 | 46,60866444 | 1,55478525 |
| Costars family protein ABRACL | ABRACL | 54,9872749 | 8,599723271 | 1,56394789 |
| N-acetylmuramoyl-L-alanine amidase | PGLYRP2 | 683,543738 | 108,4960559 | 1,58725843 |
| deoxyribose-phosphate aldolase;Deoxyribose-phosphate aldolase | DERA | 154,399393 | 24,53730752 | 1,5892101 |
| Heparin cofactor 2 | SERPIND1 | 778,224792 | 124,1214489 | 1,59493054 |
| Glycine decarboxylase (Fragment) | GLDC | 1845,84167 | 299,0521917 | 1,62014 |
| Ras-related protein Rap-1A | RAP1A | 50,4528549 | 8,179077661 | 1,62113277 |
| 14-3-3 protein beta/alpha | YWHAB | 91,8259857 | 14,89681036 | 1,62228701 |
| T-complex protein 1 subunit eta | CCT7 | 501,976904 | 81,6495455 | 1,6265598 |
| Proteasome subunit alpha type-1 | PSMA1 | 285,05994 | 46,59447202 | 1,63454998 |
| Fibronectin | FN1 | 692,952283 | 113,2844938 | 1,63480945 |
| F-box only protein 7 | FBXO7 | 1002,01796 | 164,1102788 | 1,63779778 |
| Proteasome subunit beta type-2 | PSMB2 | 669,524475 | 109,6753814 | 1,63810862 |
| Glypican-3 (Fragment);Glypican-3;Glypican-3 (Fragment);Glypican-3 | GPC3 | 73,2673225 | 12,00565329 | 1,63860953 |
| Profilin-1 | PFN1 | 736,591162 | 120,7367546 | 1,63912847 |
| Immunoglobulin kappa constant | IGKC | 46827,4742 | 7679,232811 | 1,639899 |
| WW domain binding protein 2;WW domain binding protein 2 (Fragment);WW domain binding protein 2;WW domain binding protein 2;WW domain-binding protein 2 | WBP2 | 232,501489 | 38,37634787 | 1,65058504 |
| Complement C1q A chain;Complement C1q subcomponent subunit A | C1QA | 75,7341408 | 12,5588725 | 1,65828415 |
| Serpin family G member 1;Serpin family G member 1;Plasma protease C1 inhibitor | SERPING1 | 3847,09062 | 638,6235786 | 1,66001699 |
| Immunoglobulin heavy variable 1-18 | IGHV1-18 | 182,959952 | 30,3856487 | 1,66078141 |
| Kininogen-1 | KNG1 | 7016,0373 | 1165,847105 | 1,66168886 |
| Thymosin beta-4 | TMSB4X | 85,7050461 | 14,24595136 | 1,66220684 |
| Sorbitol dehydrogenase | SORD | 126,289928 | 21,00064698 | 1,66289167 |
| Leukotriene A-4 hydrolase | LTA4H | 378,40835 | 62,92760481 | 1,66295497 |
| Calponin (Fragment);Calponin;Calponin;Calponin;Calponin-2 | CNN2 | 59,8221481 | 9,951048205 | 1,6634388 |
| Glutathione peroxidase 1 | GPX1 | 357,988147 | 59,60828288 | 1,66509096 |
| F-box protein 27 (Fragment) | FBXO27 | 51,0588455 | 8,502419554 | 1,6652197 |
| T-complex protein 1 subunit zeta | CCT6A | 175,081021 | 29,23370582 | 1,66972443 |
| 26S proteasome non-ATPase regulatory subunit 12 | PSMD12 | 134,285678 | 22,42492192 | 1,66994145 |
| Glutathione S-transferase theta-1 | GSTT1 | 78,7746902 | 13,24030013 | 1,68078098 |
| Dipeptidyl peptidase 3 | DPP3 | 162,945488 | 27,42095582 | 1,68283002 |
| Enolase-phosphatase 1;Enolase-phosphatase E1 | ENOPH1 | 21,3095276 | 3,611916314 | 1,69497719 |
| Complement component C9 | C9 | 1221,36429 | 207,7442356 | 1,70091952 |
| Ras-related protein Rap-1b | RAP1B | 425,314996 | 72,7632365 | 1,71080816 |
| Immunoglobulin lambda variable 1-36 | IGLV1-36 | 143,93381 | 24,73443709 | 1,71845914 |
| CYFIP related Rac1 interactor B;CYFIP related Rac1 interactor B (Fragment);CYFIP related Rac1 interactor B (Fragment);CYFIP related Rac1 interactor B (Fragment);CYFIP related Rac1 interactor B (Fragment);CYFIP-related Rac1 interactor B | CYRIB | 54,7041374 | 9,423753055 | 1,72267647 |
| Proliferation-associated protein 2G4 | PA2G4 | 519,87475 | 89,65095755 | 1,72447224 |
| RuvB-like 1 | RUVBL1 | 41,9241879 | 7,234942834 | 1,72572045 |
| Cysteine and glycine-rich protein 1 | CSRP1 | 37,1520142 | 6,420769635 | 1,72824267 |
| AP-2 complex subunit beta | AP2B1 | 28,4968086 | 4,934653042 | 1,73165112 |
| Heat shock protein HSP 90-alpha | HSP90AA1 | 344,480273 | 59,66913273 | 1,73214948 |
| Cell division control protein 42 homolog | CDC42 | 238,081473 | 41,30940457 | 1,7350953 |
| Major histocompatibility complex, class II, DQ beta 2;Major histocompatibility complex, class II, DQ beta 2 (Fragment);Major histocompatibility complex, class II, DQ beta 2 (Fragment);Major histocompatibility complex, class II, DQ beta 2 (Fragment);Major histocompatibility complex, class II, DQ beta 2 (Fragment);Major histocompatibility complex, class II, DQ beta 2 (Fragment);Major histocompatibility complex, class II, DQ beta 2 (Fragment);Major histocompatibility complex, class II, DQ beta 2 (Fragment);Major histocompatibility complex, class II, DQ beta 2;Major histocompatibility complex, class II, DQ beta 2 (Fragment);HLA class II histocompatibility antigen, DQ beta 2 chain;Major histocompatibility complex, class II, DQ beta 2;Major histocompatibility complex, class II, DQ beta 2 | HLA-DQB2 | 532,713409 | 92,79811781 | 1,74198952 |
| Carboxypeptidase N subunit 2 | CPN2 | 495,176099 | 86,53742033 | 1,74760899 |
| Triokinase/FMN cyclase | TKFC | 96,2481094 | 16,83840784 | 1,74947933 |
| Small glutamine rich tetratricopeptide repeat co-chaperone alpha;Small glutamine rich tetratricopeptide repeat co-chaperone alpha;Small glutamine rich tetratricopeptide repeat co-chaperone alpha;Small glutamine rich tetratricopeptide repeat co-chaperone alpha;Small glutamine rich tetratricopeptide repeat co-chaperone alpha;Small glutamine rich tetratricopeptide repeat co-chaperone alpha (Fragment);Small glutamine-rich tetratricopeptide repeat-containing protein alpha | SGTA | 113,242068 | 19,82112456 | 1,75033227 |
| Antithrombin-III | SERPINC1 | 920,707581 | 161,3784299 | 1,7527653 |
| Ras-related protein Rap-2b | RAP2B | 75,8194916 | 13,29145201 | 1,75303893 |
| 26S proteasome non-ATPase regulatory subunit 10 | PSMD10 | 176,830124 | 31,08675243 | 1,75800094 |
| Protein C receptor (Fragment);Endothelial protein C receptor | PROCR | 74,7891594 | 13,15609654 | 1,75909138 |
| guanylate kinase;guanylate kinase;Guanylate kinase | GUK1 | 70,071936 | 12,34758342 | 1,76212962 |
| Delta-aminolevulinic acid dehydratase | ALAD | 2730,47734 | 481,2656366 | 1,7625696 |
| Complement component C7 | C7 | 416,693945 | 74,11648764 | 1,77867926 |
| Cathepsin G | CTSG | 199,614499 | 35,51561083 | 1,77920998 |
| 26S proteasome regulatory subunit 4 | PSMC1 | 51,5347443 | 9,221205217 | 1,78931813 |
| Coagulation factor XIII A chain | F13A1 | 78,4478455 | 14,07800157 | 1,79456829 |
| Proteasome subunit beta type-5 | PSMB5 | 96,9666122 | 17,40694695 | 1,79514851 |
| (E3-independent) E2 ubiquitin-conjugating enzyme | UBE2O | 103,806618 | 18,63901149 | 1,79555137 |
| Glutathione S-transferase;Glutathione S-transferase;Glutathione S-transferase Mu 2 | GSTM2 | 72,0317741 | 12,96525117 | 1,799935 |
| Inter-alpha-trypsin inhibitor heavy chain H3 | ITIH3 | 666,730359 | 120,0141175 | 1,80003979 |
| Perilipin 3 (Fragment);Perilipin-3 | PLIN3 | 35,5048458 | 6,392998445 | 1,80059885 |
| Alpha-2-macroglobulin | A2M | 19307,3687 | 3481,207618 | 1,80304612 |
| Serum paraoxonase/lactonase 3 | PON3 | 58,3746994 | 10,55359616 | 1,80790587 |
| Endophilin-B2 | SH3GLB2 | 67,6645294 | 12,24633621 | 1,80986055 |
| protein disulfide-isomerase;Protein disulfide-isomerase;Protein disulfide-isomerase;Protein disulfide-isomerase;Protein disulfide-isomerase;Protein disulfide-isomerase;Protein disulfide-isomerase;Protein disulfide-isomerase;Protein disulfide-isomerase;Protein disulfide-isomerase;protein disulfide-isomerase;protein disulfide-isomerase;protein disulfide-isomerase;Protein disulfide-isomerase;Protein disulfide-isomerase;protein disulfide-isomerase;Protein disulfide-isomerase | P4HB | 92,8415863 | 16,81975036 | 1,81166124 |
| Hemoglobin subunit delta (Fragment) | HBD | 271,262973 | 49,17397382 | 1,81277869 |
| Zinc-alpha-2-glycoprotein | AZGP1 | 489,397412 | 89,2450733 | 1,8235706 |
| Serum amyloid P-component | APCS | 189,710403 | 34,60746905 | 1,82422622 |
| Synaptotagmin binding cytoplasmic RNA interacting protein;Synaptotagmin binding cytoplasmic RNA interacting protein;Synaptotagmin binding cytoplasmic RNA interacting protein;Synaptotagmin binding cytoplasmic RNA interacting protein;Synaptotagmin binding cytoplasmic RNA interacting protein;Synaptotagmin binding cytoplasmic RNA interacting protein;Synaptotagmin binding cytoplasmic RNA interacting protein;Heterogeneous nuclear ribonucleoprotein Q;Heterogeneous nuclear ribonucleoprotein Q | SYNCRIP | 76,1373138 | 13,91137362 | 1,82714269 |
| Immunoglobulin heavy constant alpha 2 (A2m marker) (Fragment) | IGHA2 | 18525,8223 | 3389,458385 | 1,82958593 |
| Immunoglobulin lambda like polypeptide 5;Immunoglobulin lambda constant 1 (Fragment);Immunoglobulin lambda-like polypeptide 5;Immunoglobulin lambda constant 1 | IGLL5;IGLC1;IGLL5;IGLC1 | 738,002246 | 135,2762225 | 1,83300557 |
| Dedicator of cytokinesis 10;Dedicator of cytokinesis 10 (Fragment);Dedicator of cytokinesis 10 (Fragment);Dedicator of cytokinesis protein 10 | DOCK10 | 1449,07095 | 265,8376476 | 1,83453852 |
| Bifunctional purine biosynthesis protein ATIC | ATIC | 409,539246 | 75,16560661 | 1,83537005 |
| S-methyl-5'-thioadenosine phosphorylase | MTAP | 209,084116 | 38,58745031 | 1,84554671 |
| Platelet activating factor acetylhydrolase 1b regulatory subunit 1;Platelet-activating factor acetylhydrolase IB subunit alpha;Platelet-activating factor acetylhydrolase IB subunit alpha;Platelet-activating factor acetylhydrolase IB subunit alpha;Platelet-activating factor acetylhydrolase IB subunit beta | PAFAH1B1 | 59,6063957 | 11,01765667 | 1,84840176 |
| Protein S100-A6;Protein S100 (Fragment) | S100A6 | 123,728233 | 22,87029009 | 1,84842938 |
| PITH domain-containing protein 1;PITH domain containing 1 | PITHD1 | 32,9406326 | 6,089369766 | 1,8485892 |
| Hsp90 co-chaperone Cdc37 (Fragment);Hsp90 co-chaperone Cdc37 (Fragment);Hsp90 co-chaperone Cdc37 | CDC37 | 42,8188408 | 7,925868789 | 1,85102367 |
| C-X-C motif chemokine |  | 363,117108 | 67,33234075 | 1,85428721 |
| Charged multivesicular body protein 4A (Fragment);Charged multivesicular body protein 4A;Transmembrane 9 superfamily member;Charged multivesicular body protein 4a | CHMP4A;CHMP4A;;CHMP4A | 30,3268372 | 5,626396492 | 1,8552533 |
| Tropomyosin 3 | TPM3 | 114,545551 | 21,27537315 | 1,85737228 |
| Phosphopentomutase | PGM2 | 195,499127 | 36,33614661 | 1,85863472 |
| receptor protein-tyrosine kinase;Macrophage colony-stimulating factor 1 receptor | CSF1R | 86,4131394 | 16,13605861 | 1,8673154 |
| Proteasome subunit alpha type;Proteasome subunit alpha type-6 | PSMA6 | 312,074518 | 58,57823006 | 1,87705906 |
| Isocitrate dehydrogenase [NADP] cytoplasmic | IDH1 | 66,5304039 | 12,4923354 | 1,87768819 |
| Nuclear transport factor 2 | NUTF2 | 106,464331 | 20,08619178 | 1,88665928 |
| Alpha-1-acid glycoprotein 2 | ORM2 | 4178,73398 | 788,3860304 | 1,88666241 |
| Cullin 4A;Cullin 4A;Cullin 4B;Cullin 4B (Fragment);Cullin 4B (Fragment);Cullin 4B;Cullin 4B (Fragment);Cullin 4B (Fragment);Cullin 4B;Cullin 4B (Fragment);Cullin 4B;Cullin 4B (Fragment);Cullin 4B;Cullin 4B (Fragment);Cullin 4B;Cullin 4B;Cullin-4A;Cullin-4B | CUL4A;CUL4A;CUL4B;CUL4B;CUL4B;CUL4B;CUL4B;CUL4B;CUL4B;CUL4B;CUL4B;CUL4B;CUL4B;CUL4B;CUL4B;CUL4B;CUL4A;CUL4B | 90,1613037 | 17,13926677 | 1,90095596 |
| Proteasome subunit beta type-6 | PSMB6 | 464,60365 | 88,40366538 | 1,90277595 |
| Annexin A1;Annexin (Fragment) | ANXA1 | 50,5515724 | 9,625692255 | 1,90413311 |
| Proteasome 26S subunit ubiquitin receptor, non-ATPase 4 (Fragment);26S proteasome non-ATPase regulatory subunit 4;26S proteasome non-ATPase regulatory subunit 4 | PSMD4 | 93,5433899 | 17,85896117 | 1,90916335 |
| Xaa-Pro dipeptidase | PEPD | 185,378619 | 35,39348637 | 1,90925397 |
| ubiquitinyl hydrolase 1;Ubiquitin carboxyl-terminal hydrolase 24 | USP24 | 11,421703 | 2,18644295 | 1,91428804 |
| Insulin-like growth factor 2 | IGF2 | 92,1208603 | 17,72726583 | 1,92434871 |
| Transthyretin | TTR | 598,504755 | 115,2790348 | 1,92611728 |
| Thyroxine-binding globulin | SERPINA7 | 95,1846649 | 18,34765098 | 1,92758476 |
| X-prolyl aminopeptidase 1;Xaa-Pro aminopeptidase 1 | XPNPEP1 | 63,5205154 | 12,24412636 | 1,92758612 |
| Serine/threonine-protein phosphatase (Fragment);Serine/threonine-protein phosphatase 5 | PPP5C | 27,8323566 | 5,365491325 | 1,92778908 |
| T-complex protein 1 subunit gamma | CCT3 | 313,329819 | 60,42073065 | 1,92834282 |
| Coronin;Coronin-1C | CORO1C | 143,811076 | 27,79515596 | 1,93275488 |
| Transferrin receptor protein 1 | TFRC | 42,7058048 | 8,260970788 | 1,93439061 |
| RuvB-like helicase;RuvB-like 2 | RUVBL2 | 34,8685589 | 6,757948585 | 1,93812099 |
| AMP deaminase;AMP deaminase 3 | AMPD3 | 71,9361122 | 13,96566239 | 1,94139799 |
| Superoxide dismutase copper chaperone;Copper chaperone for superoxide dismutase | CCS | 458,779645 | 89,08133154 | 1,94170192 |
| Immunoglobulin lambda variable 1-47 | IGLV1-47 | 3050,55635 | 592,4422429 | 1,94207933 |
| Protein ARPC4-TTLL3;Actin-related protein 2/3 complex subunit 4;Actin related protein 2/3 complex subunit 4;Actin-related protein 2/3 complex subunit 4 | ARPC4-TTLL3;ARPC4-TTLL3;ARPC4;ARPC4 | 145,357584 | 28,28973972 | 1,94621697 |
| Proteasome 26S subunit, ATPase 6;Proteasome 26S subunit, ATPase 6 (Fragment);26S proteasome regulatory subunit 10B | PSMC6 | 163,55354 | 32,06829748 | 1,9607217 |
| Aldo-keto reductase family 7 member A2;Aflatoxin B1 aldehyde reductase member 2 | AKR7A2 | 186,60589 | 36,6483534 | 1,96394409 |
| Malate dehydrogenase, mitochondrial | MDH2 | 36,0249634 | 7,09295845 | 1,96890095 |
| Haptoglobin | HP | 1585,98169 | 313,1909577 | 1,9747451 |
| Multimerin 1;Multimerin-1 | MMRN1 | 41,0826622 | 8,115655549 | 1,97544539 |
| Ubiquitin-conjugating enzyme E2 variant 2 | UBE2V2 | 168,696896 | 33,32569654 | 1,97547776 |
| Acidic leucine-rich nuclear phosphoprotein 32 family member;Acidic leucine-rich nuclear phosphoprotein 32 family member A | ANP32A | 65,9957458 | 13,07144366 | 1,98064944 |
| Talin-1 | TLN1 | 402,42326 | 79,92695758 | 1,9861416 |
| E2 ubiquitin-conjugating enzyme;E2 ubiquitin-conjugating enzyme;E2 ubiquitin-conjugating enzyme (Fragment);E2 ubiquitin-conjugating enzyme (Fragment);E2 ubiquitin-conjugating enzyme;Ubiquitin-conjugating enzyme E2 D3;Ubiquitin-conjugating enzyme E2 D2 | UBE2D3;UBE2D3;UBE2D3;UBE2D2;UBE2D3;UBE2D3;UBE2D2 | 160,7155 | 31,92283691 | 1,98629484 |
| Clathrin heavy chain;Clathrin heavy chain;Clathrin heavy chain;Clathrin heavy chain;Clathrin heavy chain;Clathrin heavy chain;Clathrin heavy chain;Clathrin heavy chain;Clathrin heavy chain;Clathrin heavy chain;Clathrin heavy chain;Clathrin heavy chain;Clathrin heavy chain;Clathrin heavy chain 1 | CLTC | 102,957893 | 20,47431216 | 1,98861025 |
| Pregnancy zone protein | PZP | 157,313654 | 31,40225568 | 1,99615576 |
| 14-3-3 protein theta | YWHAQ | 80,7610794 | 16,18923582 | 2,00458388 |
| hydroxymethylbilane synthase;hydroxymethylbilane synthase;hydroxymethylbilane synthase;hydroxymethylbilane synthase;Porphobilinogen deaminase | HMBS | 574,331763 | 115,3123561 | 2,00776561 |
| Extracellular matrix protein 1 | ECM1 | 46,9436195 | 9,430488369 | 2,00889673 |
| Arginyl aminopeptidase (Fragment);Arginyl aminopeptidase (Fragment);Arginyl aminopeptidase;Arginyl aminopeptidase (Fragment);Aminopeptidase B | RNPEP | 105,641376 | 21,26918555 | 2,01333856 |
| Hemoglobin subunit theta-1 | HBQ1 | 704,029675 | 141,86609 | 2,01505839 |
| ATP-dependent 6-phosphofructokinase;ATP-dependent 6-phosphofructokinase;ATP-dependent 6-phosphofructokinase, muscle type | PFKM | 25,8399307 | 5,208226391 | 2,01557289 |
| FERM domain containing kindlin 3;FERM domain containing kindlin 3;Fermitin family homolog 3 | FERMT3 | 120,43788 | 24,28997541 | 2,01680529 |
| Tubulin alpha-1B chain | TUBA1B | 223,159317 | 45,01918313 | 2,0173562 |
| Immunoglobulin heavy variable 5-51 | IGHV5-51 | 380,775378 | 77,05390839 | 2,02360533 |
| Carbonic anhydrase 3 | CA3 | 2409,29302 | 488,3461044 | 2,02692699 |
| Isoamyl acetate-hydrolyzing esterase 1 homolog (Fragment);Isoamyl acetate-hydrolyzing esterase 1 homolog | IAH1 | 68,3599464 | 13,89298546 | 2,03232831 |
| Catalase | CAT | 8623,9668 | 1754,268863 | 2,03417859 |
| Dynactin subunit 2;Dynactin subunit 2;Dynactin subunit 2;Dynactin subunit 2 (Fragment);Dynactin subunit 2 | DCTN2 | 9,50360079 | 1,948320979 | 2,05008714 |
| Eukaryotic translation initiation factor 4B | EIF4B | 30,2703091 | 6,255108854 | 2,06641724 |
| Immunoglobulin heavy variable 1-8 | IGHV1-8 | 41,8560905 | 8,667679307 | 2,07082869 |
| Importin 5 (Fragment);Importin 5 (Fragment);Importin 5 (Fragment);Importin-5 | IPO5 | 92,6340744 | 19,35585775 | 2,08949653 |
| Filamin-A;Filamin A | FLNA | 313,413855 | 65,56001483 | 2,09180334 |
| Complement component C8 gamma chain | C8G | 699,702112 | 146,5231405 | 2,09407887 |
| Vitronectin | VTN | 3199,4895 | 671,7469015 | 2,09954401 |
| Actin gamma 1;Actin, cytoplasmic 1;Actin, cytoplasmic 2 | ACTG1;ACTB;ACTG1 | 7602,16953 | 1612,571101 | 2,12119855 |
| 14-3-3 protein gamma | YWHAG | 63,7275314 | 13,57015726 | 2,12940262 |
| Immunoglobulin heavy variable 4-61;Immunoglobulin heavy variable 4-39;Immunoglobulin heavy variable 4-59;Immunoglobulin heavy variable 4-34;Immunoglobulin heavy variable 4-30-4;Immunoglobulin heavy variable 4-38-2 | IGHV4-61;IGHV4-39;IGHV4-59;IGHV4-34;IGHV4-30-4;IGHV4-38-2 | 292,145142 | 62,37771789 | 2,13516191 |
| Voltage-dependent L-type calcium channel subunit beta-2;Calcium voltage-gated channel auxiliary subunit beta 4;Calcium voltage-gated channel auxiliary subunit beta 4;Calcium voltage-gated channel auxiliary subunit beta 4;Calcium voltage-gated channel auxiliary subunit beta 4;Calcium voltage-gated channel auxiliary subunit beta 4;Calcium voltage-gated channel auxiliary subunit beta 4;Voltage-dependent L-type calcium channel subunit beta-3;Calcium voltage-gated channel auxiliary subunit beta 4;Voltage-dependent L-type calcium channel subunit beta-3;Calcium voltage-gated channel auxiliary subunit beta 4;Voltage-dependent L-type calcium channel subunit beta-3 (Fragment);Calcium voltage-gated channel auxiliary subunit beta 4;Voltage-dependent L-type calcium channel subunit beta-2 (Fragment);Voltage-dependent L-type calcium channel subunit beta-2 (Fragment);Voltage-dependent L-type calcium channel subunit beta-2;Voltage-dependent L-type calcium channel subunit beta-2;Voltage-dependent L-type calcium channel subunit beta-2;Calcium voltage-gated channel auxiliary subunit beta 4;Calcium voltage-gated channel auxiliary subunit beta 3 (Fragment);Calcium voltage-gated channel auxiliary subunit beta 3 (Fragment);Calcium voltage-gated channel auxiliary subunit beta 4;Voltage-dependent L-type calcium channel subunit beta-4;Voltage-dependent L-type calcium channel subunit beta-3;Voltage-dependent L-type calcium channel subunit beta-1;Voltage-dependent L-type calcium channel subunit beta-2 | CACNB2;CACNB4;CACNB4;CACNB4;CACNB4;CACNB4;CACNB4;CACNB4;CACNB4;CACNB4;CACNB4;CACNB4;CACNB4;CACNB2;CACNB2;CACNB2;CACNB2;CACNB2;CACNB4;CACNB3;CACNB3;CACNB4;CACNB4;CACNB3;CACNB1;CACNB2 | 669,160437 | 143,3255878 | 2,14187181 |
| Coactosin like F-actin binding protein 1;Coactosin-like protein | COTL1 | 41,1421906 | 8,860737763 | 2,15368643 |
| NudC domain containing 2;NudC domain-containing protein 2 | NUDCD2 | 21,6155384 | 4,660614084 | 2,15614064 |
| NEDD8-activating enzyme E1 regulatory subunit | NAE1 | 40,0553818 | 8,666499563 | 2,16362925 |
| Pleckstrin | PLEK | 85,8193542 | 18,57615087 | 2,16456428 |
| Apolipoprotein B;Apolipoprotein B-100 | APOB | 3070,66362 | 668,4559889 | 2,1769105 |
| Carboxymethylenebutenolidase homolog | CMBL | 266,076859 | 57,9340577 | 2,17734296 |
| Cytosolic non-specific dipeptidase | CNDP2 | 51,6922813 | 11,26053243 | 2,17837792 |
| Dynactin subunit 1;Dynactin subunit 1;Uncharacterized protein (Fragment);Dynactin subunit 1;Dynactin subunit 1 | DCTN1;DCTN1;;DCTN1;DCTN1 | 32,7955318 | 7,170886337 | 2,1865437 |
| Ras suppressor protein 1 | RSU1 | 230,660007 | 51,03503586 | 2,21256543 |
| Vitamin K-dependent protein C;Vitamin K-dependent protein C (Fragment);Vitamin K-dependent protein C | PROC | 59,1092834 | 13,09176018 | 2,21483994 |
| Chromosome 6 open reading frame 55, isoform CRA_b;Vacuolar protein sorting-associated protein VTA1 homolog | VTA1 | 220,379343 | 49,04576031 | 2,22551532 |
| Hemoglobin subunit mu | HBM | 252,328644 | 56,30020764 | 2,23122539 |
| Heparin binding growth factor;Hepatoma-derived growth factor | HDGF | 31,7722935 | 7,097899262 | 2,23399021 |
| Transport and golgi organization 2 homolog (Fragment);Transport and Golgi organization protein 2 homolog | TANGO2 | 175,047105 | 39,12697194 | 2,23522531 |
| Protein S100-A4 | S100A4 | 267,916815 | 59,9700036 | 2,23838148 |
| CRK proto-oncogene, adaptor protein;Adapter molecule crk | CRK | 20,116659 | 4,503183473 | 2,23853448 |
| Tubulin beta-1 chain | TUBB1 | 146,924069 | 32,94944255 | 2,24261707 |
| 4a-hydroxytetrahydrobiopterin dehydratase (Fragment);Pterin-4-alpha-carbinolamine dehydratase 2 | PCBD2 | 67,5334396 | 15,19141626 | 2,2494658 |
| Protein-glutamine gamma-glutamyltransferase 2 | TGM2 | 314,779935 | 71,06967954 | 2,25775762 |
| Putative peptidyl-tRNA hydrolase PTRHD1 | PTRHD1 | 8,07641468 | 1,823484989 | 2,2577902 |
| Glutathione S-transferase P | GSTP1 | 1128,85372 | 256,3786519 | 2,27114149 |
| Immunoglobulin heavy variable 3-49 | IGHV3-49 | 68,0424889 | 15,47134377 | 2,27377687 |
| Tubulin alpha-4A chain | TUBA4A | 337,061841 | 76,73138971 | 2,27647809 |
| Complement C1q C chain;Complement C1q subcomponent subunit C | C1QC | 310,875656 | 70,8210962 | 2,2781165 |
| Coagulation factor XII | F12 | 365,107928 | 83,21778884 | 2,27926546 |
| Macrophage migration inhibitory factor | MIF | 843,724817 | 192,6965169 | 2,28387874 |
| Translin | TSN | 46,5170944 | 10,64999699 | 2,28948027 |
| Serum paraoxonase/arylesterase 1 | PON1 | 2382,30183 | 545,5833684 | 2,29015216 |
| SH3 domain-binding glutamic acid-rich-like protein 3 | SH3BGRL3 | 176,190887 | 40,37676078 | 2,29164865 |
| RAB6A, member RAS oncogene family;RAB6A, member RAS oncogene family (Fragment);Ras-related protein Rab-6A | RAB6A | 16,9721642 | 3,89378935 | 2,29422088 |
| Tyrosine--tRNA ligase;Tyrosine--tRNA ligase, cytoplasmic | YARS1 | 49,9886368 | 11,48574209 | 2,2976706 |
| Immunoglobulin kappa variable 3D-11;Immunoglobulin kappa variable 3-11 | IGKV3D-11;IGKV3-11 | 5504,19751 | 1267,119701 | 2,30209708 |
| V-type proton ATPase subunit B, brain isoform | ATP6V1B2 | 46,3778343 | 10,74745316 | 2,31736848 |
| Fructosamine-3-kinase | FN3K | 124,724606 | 29,03508384 | 2,3279355 |
| Cysteine--tRNA ligase, cytoplasmic;Cysteine--tRNA ligase, cytoplasmic | CARS1 | 33,3595226 | 7,770361212 | 2,3292783 |
| peptidylprolyl isomerase (Fragment) | PPIG | 50,4899338 | 11,78003263 | 2,3331448 |
| Myosin light chain 4 (Fragment);Myosin light chain 3;Myosin light chain 4 (Fragment);Myosin light chain 4;Myosin light chain 4 (Fragment);Myosin light chain 3;Myosin light chain 4 | MYL4;MYL3;MYL4;MYL4;MYL4;MYL3;MYL4 | 29,1030945 | 6,816282682 | 2,34211612 |
| Malignant T-cell-amplified sequence 1 | MCTS1 | 204,025217 | 47,83984785 | 2,34480073 |
| Myosin light chain 6;Myosin light chain 6;Myosin light chain 6;Myosin light chain 6;Myosin light polypeptide 6;Myosin light polypeptide 6 | MYL6 | 129,911409 | 30,48212922 | 2,34637816 |
| Gamma-enolase | ENO2 | 86,5284012 | 20,38533342 | 2,35591241 |
| Galectin-3-binding protein | LGALS3BP | 54,0147255 | 12,73957534 | 2,35853746 |
| Complement factor H related 1;Complement factor H-related protein 1 | CFHR1 | 49,5333893 | 11,72102242 | 2,36628718 |
| DAZ associated protein 1;DAZ associated protein 1 (Fragment);DAZ associated protein 1;DAZ-associated protein 1 | DAZAP1 | 59,7537987 | 14,15503424 | 2,36889278 |
| ubiquitinyl hydrolase 1;ubiquitinyl hydrolase 1;ubiquitinyl hydrolase 1;Ubiquitin specific peptidase 9 X-linked (Fragment);Ubiquitin carboxyl-terminal hydrolase 9X | USP9X | 14,2280552 | 3,380079252 | 2,3756439 |
| Immunoglobulin kappa variable 3-15 | IGKV3-15 | 3061,72813 | 727,6478386 | 2,37659194 |
| Pyruvate kinase PKLR | PKLR | 328,589777 | 78,22957916 | 2,38076729 |
| Heterogeneous nuclear ribonucleoprotein H1;Heterogeneous nuclear ribonucleoprotein H1;Heterogeneous nuclear ribonucleoprotein H1;Heterogeneous nuclear ribonucleoprotein H1 (Fragment);Heterogeneous nuclear ribonucleoprotein H1 (Fragment);Heterogeneous nuclear ribonucleoprotein H1 (Fragment);Heterogeneous nuclear ribonucleoprotein H1 (Fragment);Heterogeneous nuclear ribonucleoprotein H1;Heterogeneous nuclear ribonucleoprotein H1;Heterogeneous nuclear ribonucleoprotein H1;Heterogeneous nuclear ribonucleoprotein H;Heterogeneous nuclear ribonucleoprotein H2 | HNRNPH1;HNRNPH1;HNRNPH1;HNRNPH1;HNRNPH1;HNRNPH1;HNRNPH1;HNRNPH1;HNRNPH1;HNRNPH1;HNRNPH1;HNRNPH2 | 43,9317795 | 10,48086208 | 2,38571308 |
| Protein disulfide-isomerase A3;Protein disulfide-isomerase A3;Protein disulfide-isomerase;Protein disulfide-isomerase A3 | PDIA3 | 15,960005 | 3,81741299 | 2,39186203 |
| Phospholysine phosphohistidine inorganic pyrophosphate phosphatase | LHPP | 82,9400818 | 19,86182023 | 2,39471915 |
| CDGSH iron sulfur domain 2;CDGSH iron-sulfur domain-containing protein 2 | CISD2 | 164,073843 | 39,5989654 | 2,41348436 |
| Eukaryotic translation initiation factor 2 subunit 3 | EIF2S3 | 16,718751 | 4,061338691 | 2,42921178 |
| 14-3-3 protein eta | YWHAH | 36,2869709 | 8,830794703 | 2,4335993 |
| Tumor protein p53 inducible protein 3 (Fragment);Quinone oxidoreductase PIG3 | TP53I3 | 53,3571182 | 13,01143035 | 2,43855568 |
| Immunoglobulin kappa variable 4-1 | IGKV4-1 | 209,072134 | 51,02431662 | 2,44051255 |
| Aspartyl aminopeptidase | DNPEP | 51,4924839 | 12,59219745 | 2,44544378 |
| COP9 signalosome complex subunit 3 | COPS3 | 31,3745304 | 7,680033533 | 2,44785609 |
| Complement component C8 beta chain | C8B | 305,364163 | 74,8825967 | 2,45223919 |
| 26S proteasome non-ATPase regulatory subunit 7 | PSMD7 | 15,1492184 | 3,720297535 | 2,45576863 |
| Alpha-synuclein | SNCA | 2598,11038 | 639,294003 | 2,4606114 |
| Scaffold protein ILK | ILK | 26,3413857 | 6,510041261 | 2,47141184 |
| O-phosphoseryl-tRNA(Sec) selenium transferase;O-phosphoseryl-tRNA(Sec) selenium transferase;O-phosphoseryl-tRNA(Sec) selenium transferase (Fragment);O-phosphoseryl-tRNA(Sec) selenium transferase | SEPSECS | 8,54876404 | 2,118778127 | 2,47846135 |
| Vinculin | VCL | 167,910938 | 41,83250822 | 2,49135099 |
| Complement C8 alpha chain;Complement C8 alpha chain;Complement C8 alpha chain;Complement component C8 alpha chain | C8A | 38,2854458 | 9,553055312 | 2,49521851 |
| NEDD8-conjugating enzyme Ubc12 | UBE2M | 30,588147 | 7,658665095 | 2,50380159 |
| V-type proton ATPase catalytic subunit A | ATP6V1A | 49,6004135 | 12,44930172 | 2,50991894 |
| Uncharacterized protein (Fragment);Ras-related protein Rab-5C | ;RAB5C | 13,0309668 | 3,276013368 | 2,51402173 |
| Immunoglobulin kappa variable 2-28;Immunoglobulin kappa variable 2-40;Immunoglobulin kappa variable 2-40 (Fragment);Immunoglobulin kappa variable 2-40;Immunoglobulin kappa variable 2D-40;Immunoglobulin kappa variable 2D-28 | IGKV2-28;IGKV2-40;IGKV2-40;IGKV2-40;IGKV2D-40;IGKV2D-28 | 562,353186 | 141,4611041 | 2,51552063 |
| Cadherin 5;Cadherin-5 | CDH5 | 133,212746 | 33,62667636 | 2,5242837 |
| Actin-related protein 3 | ACTR3 | 24,88582 | 6,321705348 | 2,54028412 |
| Osteoclast-stimulating factor 1 | OSTF1 | 24,8770319 | 6,324741779 | 2,54240209 |
| LIM zinc finger domain containing 3;LIM zinc finger domain containing 1;LIM and senescent cell antigen-like-containing domain protein 1 | LIMS3;LIMS1;LIMS1 | 223,315286 | 56,79876766 | 2,54343393 |
| Ras-related protein Rab-14;Ras-related protein Rab-14;Ras-related protein Rab-14 (Fragment) | RAB14 | 13,2259705 | 3,366943549 | 2,54570623 |
| Immunoglobulin kappa variable 1-12;Immunoglobulin kappa variable 1-39;Immunoglobulin kappa variable 1D-12;Immunoglobulin kappa variable 1D-39 | IGKV1-12;IGKV1-39;IGKV1D-12;IGKV1D-39 | 346,182809 | 88,35890402 | 2,552377 |
| ATP-dependent RNA helicase (Fragment);RNA helicase;ATP-dependent RNA helicase (Fragment);ATP-dependent RNA helicase (Fragment);ATP-dependent RNA helicase (Fragment);ATP-dependent RNA helicase (Fragment);RNA helicase (Fragment);Eukaryotic initiation factor 4A-I | EIF4A1 | 157,056311 | 40,18101175 | 2,5583825 |
| BRO1 domain-containing protein BROX | BROX | 22,10289 | 5,656865025 | 2,55933275 |
| Zyxin | ZYX | 83,3888077 | 21,34845375 | 2,5601102 |
| 14 kDa phosphohistidine phosphatase | PHPT1 | 35,0342529 | 9,000645647 | 2,56909878 |
| Immunoglobulin kappa variable 3-20 | IGKV3-20 | 2439,71912 | 627,8189028 | 2,57332452 |
| Putative hydrolase DDAH2 | DDAH2 | 32,9901238 | 8,511815201 | 2,58011011 |
| Immunoglobulin heavy variable 3/OR16-12 (non-functional) (Fragment) | IGHV3OR16-12 | 582,946661 | 150,6677582 | 2,58458909 |
| Fibulin-1 | FBLN1 | 66,7631683 | 17,29402025 | 2,59035344 |
| Ig-like domain-containing protein (Fragment);Immunoglobulin heavy variable 3-23;Immunoglobulin heavy variable 3-30;Immunoglobulin heavy variable 3-30-5 | ;IGHV3-23;IGHV3-30;IGHV3-30-5 | 572,514197 | 148,9602376 | 2,60186103 |
| 2-iminobutanoate/2-iminopropanoate deaminase (Fragment);2-iminobutanoate/2-iminopropanoate deaminase (Fragment);2-iminobutanoate/2-iminopropanoate deaminase | RIDA | 37,2172852 | 9,731932018 | 2,61489573 |
| FAS-associated factor 1 | FAF1 | 20,7366352 | 5,428550897 | 2,61785523 |
| Thrombospondin-1 | THBS1 | 136,811809 | 35,91196073 | 2,62491674 |
| Alpha-hemoglobin-stabilizing protein | AHSP | 881,114771 | 231,6116427 | 2,62862059 |
| tryptophan--tRNA ligase (Fragment);Tryptophan--tRNA ligase, cytoplasmic | WARS1 | 109,847891 | 29,00276368 | 2,64026586 |
| Gelsolin (Fragment) | GSN | 14,0735069 | 3,72603775 | 2,64755457 |
| Pyrroline-5-carboxylate reductase;Pyrroline-5-carboxylate reductase 3;Pyrroline-5-carboxylate reductase 3 | PYCR3 | 26,4315201 | 7,002360795 | 2,64924634 |
| Proteasome subunit beta type-4 | PSMB4 | 137,73681 | 36,53819836 | 2,6527548 |
| Complement C1q B chain (Fragment);Complement C1q B chain;Complement C1q B chain;Complement C1q B chain;Complement C1q subcomponent subunit B | C1QB | 56,2075699 | 14,94742553 | 2,65932606 |
| Probable non-functional immunoglobulin heavy variable 3-35 | IGHV3-35 | 297,021152 | 79,03370382 | 2,66087796 |
| Ribonucleotide reductase catalytic subunit M1;Ribonucleoside-diphosphate reductase large subunit | RRM1 | 25,3279404 | 6,750259547 | 2,66514349 |
| Caveolae-associated protein 2 | CAVIN2 | 73,2976448 | 19,55932398 | 2,66847919 |
| dTMP kinase (Fragment);Thymidylate kinase | DTYMK | 60,8698242 | 16,27052015 | 2,67300265 |
| Septin-2;Septin 2 (Fragment);Septin-2 | SEPTIN2 | 45,4199303 | 12,27550867 | 2,70267008 |
| Immunoglobulin kappa variable 1-27;Immunoglobulin kappa variable 1-8;Immunoglobulin kappa variable 1-9 | IGKV1-27;IGKV1-8;IGKV1-9 | 537,010168 | 146,6055658 | 2,73003333 |
| Inorganic pyrophosphatase | PPA1 | 202,588046 | 55,78092427 | 2,75341637 |
| Bis(5'-nucleosyl)-tetraphosphatase [asymmetrical] | NUDT2 | 19,6090279 | 5,420734348 | 2,76440748 |
| Immunoglobulin heavy constant alpha 1 | IGHA1 | 7003,57881 | 1943,734712 | 2,77534496 |
| Proteasome subunit beta type-3 (Fragment);Proteasome subunit beta type-3 | PSMB3 | 112,894406 | 31,4993522 | 2,79016059 |
| Beta-Ala-His dipeptidase | CNDP1 | 117,668808 | 32,84186243 | 2,79104233 |
| Transgelin-2;Transgelin 2 (Fragment) | TAGLN2 | 229,191882 | 64,22459477 | 2,80221944 |
| Ubiquitin-fold modifier-conjugating enzyme 1 | UFC1 | 284,735898 | 80,23617978 | 2,81791584 |
| Serine/threonine-protein phosphatase 2A 56 kDa regulatory subunit;Serine/threonine-protein phosphatase 2A 56 kDa regulatory subunit delta isoform | PPP2R5D | 53,4238045 | 15,11901902 | 2,83001541 |
| PDZ and LIM domain protein 1 | PDLIM1 | 46,8084778 | 13,28338461 | 2,83781598 |
| 3'(2'),5'-bisphosphate nucleotidase 1;3'(2'), 5'-bisphosphate nucleotidase 1 (Fragment);3'(2'), 5'-bisphosphate nucleotidase 1 (Fragment);3'(2'), 5'-bisphosphate nucleotidase 1 (Fragment);3'(2'),5'-bisphosphate nucleotidase 1 | BPNT1 | 27,7524155 | 7,887693239 | 2,84216458 |
| Glutaredoxin-1 | GLRX | 376,760168 | 107,9782481 | 2,86596772 |
| Complement component C6 | C6 | 305,534079 | 87,58605227 | 2,86665411 |
| Hemoglobin subunit epsilon | HBE1 | 8979,0457 | 2574,175446 | 2,86686975 |
| Diphosphomevalonate decarboxylase (Fragment);Diphosphomevalonate decarboxylase | MVD | 64,1990776 | 18,47709243 | 2,87809313 |
| Copine 1;Copine 1;Copine 1;Copine 1 (Fragment);Copine 1 (Fragment);Copine 1 (Fragment);Copine 1 (Fragment);Copine 1 (Fragment);Copine 1 (Fragment);Copine 1 (Fragment);Copine 1 (Fragment);Copine 1 (Fragment);Copine-1 | CPNE1 | 122,605992 | 35,52491934 | 2,89748639 |
| Immunoglobulin kappa variable 3D-20 | IGKV3D-20 | 1025,80848 | 298,2684966 | 2,90764312 |
| Regulator of G-protein signaling 10 | RGS10 | 37,2204624 | 10,93161464 | 2,93699055 |
| Probable non-functional immunoglobulinn kappa variable 1-37;Probable non-functional immunoglobulinn kappa variable 1D-37 | IGKV1-37;IGKV1D-37 | 136,117743 | 40,01355539 | 2,93962819 |
| Phosphatidylinositol-glycan-specific phospholipase D | GPLD1 | 265,122263 | 78,05264932 | 2,94402471 |
| Methylthioribose-1-phosphate isomerase | MRI1 | 47,1115925 | 13,88994245 | 2,94830671 |
| Ubiquitin B;Ubiquitin C (Fragment);Ubiquitin C (Fragment);Ubiquitin C (Fragment);Ubiquitin C (Fragment);Ubiquitin C (Fragment);Ubiquitin C (Fragment);Ubiquitin C (Fragment);Ubiquitin B (Fragment);Ubiquitin B (Fragment);Ubiquitin-40S ribosomal protein S27a (Fragment);Polyubiquitin-B;Polyubiquitin-C;Ubiquitin-ribosomal protein eS31 fusion protein;Ubiquitin-ribosomal protein eL40 fusion protein;UBC protein | UBB;UBC;UBC;UBC;UBC;UBC;UBC;UBC;UBB;UBB;RPS27A;UBB;UBC;RPS27A;UBA52;UBC | 503,15105 | 148,9381339 | 2,96010778 |
| Immunoglobulin kappa variable 1-33 | IGKV1-33 | 164,970576 | 48,91920858 | 2,96532932 |
| COP9 signalosome subunit 2;COP9 signalosome complex subunit 2 | COPS2 | 19,4020927 | 5,758729138 | 2,96809691 |
| Triosephosphate isomerase | TPI1 | 1207,0646 | 359,0107117 | 2,97424605 |
| Immunoglobulin kappa variable 6-21 | IGKV6-21 | 22,3759602 | 6,659102316 | 2,9760074 |
| Tropomyosin 1 | TPM1 | 359,29801 | 107,6799594 | 2,99695396 |
| Apolipoprotein M | APOM | 269,815512 | 81,49735485 | 3,02048441 |
| CTP synthase;CTP synthase;CTP synthase 1;CTP synthase;CTP synthase;CTP synthase;CTP synthase;CTP synthase 1 | CTPS1 | 28,5242332 | 8,71427993 | 3,05504441 |
| Phosphoribosyl pyrophosphate synthetase associated protein 1 (Fragment);Phosphoribosyl pyrophosphate synthase-associated protein 1 | PRPSAP1 | 50,5162422 | 15,43434323 | 3,05532291 |
| Dihydropteridine reductase | QDPR | 110,165224 | 33,77649492 | 3,06598523 |
| Septin 7;Septin;Septin;Septin-7 | SEPTIN7 | 20,9521515 | 6,428344143 | 3,06810694 |
| Apolipoprotein L1 | APOL1 | 212,098773 | 65,28162479 | 3,0778879 |
| Neutrophil elastase | ELANE | 32,4022984 | 9,978969179 | 3,07971029 |
| Complement C4-A | C4A | 2953,95747 | 918,1021613 | 3,10804123 |
| Phenylethanolamine N-methyltransferase | PNMT | 574,744128 | 179,0373442 | 3,11507914 |
| Beta-2-glycoprotein 1 | APOH | 2794,81008 | 870,9219564 | 3,11621159 |
| Alanine aminotransferase 1;Alanine aminotransferase 2 | GPT;GPT2 | 45,3932663 | 14,23373407 | 3,13564879 |
| Immunoglobulin kappa variable 1-5 | IGKV1-5 | 683,459485 | 214,4823115 | 3,13818619 |
| Protein 4.1 | EPB41 | 85,3022995 | 26,87076541 | 3,15006343 |
| RUN domain-containing protein 3A | RUNDC3A | 230,344852 | 72,58766069 | 3,15126038 |
| Endothelin receptor type B | EDNRB | 62,406266 | 19,67141556 | 3,15215391 |
| Coronin-1A | CORO1A | 34,8523903 | 11,04468513 | 3,16898928 |
| Adipose-secreted signaling protein | ADISSP | 167,535385 | 53,31281159 | 3,18218218 |
| Elongation factor 2 | EEF2 | 108,370998 | 34,53720941 | 3,18694208 |
| Plasma serine protease inhibitor | SERPINA5 | 29,4872675 | 9,403682091 | 3,18906528 |
| Ubiquitin-fold modifier 1 (Fragment);Ubiquitin-fold modifier 1 | UFM1 | 249,591498 | 79,93348565 | 3,20257245 |
| Immunoglobulin heavy variable 1-69D;Immunoglobulin heavy variable 1-69 | IGHV1-69D;IGHV1-69 | 573,078833 | 183,7589901 | 3,20652203 |
| Joining chain of multimeric IgA and IgM (Fragment);Immunoglobulin J chain | JCHAIN | 1030,7061 | 331,39585 | 3,21523129 |
| Myeloblastin;Proteinase 3 | PRTN3 | 388,279538 | 125,0962072 | 3,22180787 |
| DnaJ heat shock protein family (Hsp40) member A4;DnaJ heat shock protein family (Hsp40) member A4;DnaJ heat shock protein family (Hsp40) member A4;DnaJ heat shock protein family (Hsp40) member A4;DnaJ heat shock protein family (Hsp40) member A4;DnaJ homolog subfamily A member 4 | DNAJA4 | 28,6008724 | 9,219275332 | 3,22342452 |
| Alpha-globin | HBA1 | 175,078049 | 56,46713225 | 3,22525483 |
| Aldo-keto reductase family 1 member A1 | AKR1A1 | 95,2798874 | 30,84357093 | 3,23715443 |
| Immunoglobulin lambda variable 3-10 | IGLV3-10 | 87,6839767 | 28,57563489 | 3,25893464 |
| Actin-related protein 2 | ACTR2 | 18,3914633 | 5,997872129 | 3,26122616 |
| Ubiquitin like 4A;Ubiquitin-like protein 4A;Ubiquitin-like protein 4A | UBL4A | 5,92594271 | 1,940954351 | 3,27535119 |
| YKT6 v-SNARE homolog;YKT6 v-SNARE homolog;YKT6 v-SNARE homolog;Synaptobrevin homolog YKT6 | YKT6 | 22,5268448 | 7,384772955 | 3,27821007 |
| Cytidine/uridine monophosphate kinase 1;UMP-CMP kinase;UMP-CMP kinase;Cytidine/uridine monophosphate kinase 1 | CMPK1 | 207,497775 | 68,15503817 | 3,28461537 |
| Ig-like domain-containing protein |  | 311,355206 | 102,3232433 | 3,28638292 |
| RNA helicase;Eukaryotic initiation factor 4A-II | EIF4A2 | 46,1948212 | 15,18194255 | 3,28650315 |
| Ficolin-3 | FCN3 | 140,80271 | 46,59566371 | 3,30928742 |
| NUBP iron-sulfur cluster assembly factor 1, cytosolic (Fragment);NUBP iron-sulfur cluster assembly factor 1, cytosolic (Fragment);Cytosolic Fe-S cluster assembly factor NUBP1 | NUBP1 | 135,87341 | 45,3752006 | 3,33952026 |
| Ubiquitin-associated domain-containing protein 1 | UBAC1 | 39,1128754 | 13,07516342 | 3,34293076 |
| Immunoglobulin heavy constant gamma 2 | IGHG2 | 21695,7174 | 7260,574018 | 3,3465471 |
| Immunoglobulin kappa variable 1-17 | IGKV1-17 | 68,8476578 | 23,09609249 | 3,35466641 |
| Fumarylacetoacetase | FAH | 296,309375 | 99,95533009 | 3,37334349 |
| cytochrome-b5 reductase;NADH-cytochrome b5 reductase;Cytochrome b5 reductase 3 (Fragment);cytochrome-b5 reductase;cytochrome-b5 reductase (Fragment);cytochrome-b5 reductase;cytochrome-b5 reductase;NADH-cytochrome b5 reductase;cytochrome-b5 reductase (Fragment);NADH-cytochrome b5 reductase 3 | CYB5R3 | 3436,89839 | 1162,854551 | 3,38344175 |
| Immunoglobulin heavy variable 3-64D | IGHV3-64D | 40,883543 | 13,8941533 | 3,39847094 |
| Apolipoprotein C-I (Fragment);Apolipoprotein C-I | APOC1 | 813,138098 | 277,2621871 | 3,40977981 |
| Actin alpha 2, smooth muscle;Actin alpha 2, smooth muscle;Actin, aortic smooth muscle | ACTA2 | 52,1814919 | 17,87147458 | 3,42486846 |
| Coagulation factor V | F5 | 51,1655304 | 17,5460239 | 3,42926649 |
| Ubiquitin-like-conjugating enzyme ATG3 | ATG3 | 11,4161525 | 3,941365004 | 3,452446 |
| L-selectin | SELL | 30,2353016 | 10,44862808 | 3,45577108 |
| Coagulation factor X | F10 | 74,74217 | 25,94333643 | 3,47104405 |
| Histone H2A;Histone H2A;Histone H2A;Histone H2A (Fragment);Histone H2A type 1-B/E;Histone H2A type 1;Histone H2AX;Histone H2A type 1-D;Histone H2A type 3;Histone H2A type 2-B;Histone H2A type 1-C;Histone H2A type 1-H;Histone H2A type 1-J;Histone H2A.J | hCG_2039566;;;H2AJ;H2AC4;H2AC11;H2AX;H2AC7;H2AC25;H2AC21;H2AC6;H2AC12;H2AC14;H2AJ | 116,558835 | 40,64612846 | 3,48717697 |
| Four and a half LIM domains 1 (Fragment);Four and a half LIM domains 1 (Fragment);Four and a half LIM domains 1 (Fragment);Four and a half LIM domains 1 (Fragment);Four and a half LIM domains 1;Four and a half LIM domains protein 1;Four and a half LIM domains 1 (Fragment);Four and a half LIM domains 1 (Fragment);Four and a half LIM domains 1 (Fragment);Four and a half LIM domains 1 (Fragment);Four and a half LIM domains 1;Four and a half LIM domains 1 (Fragment);Four and a half LIM domains 1 (Fragment) | FHL1 | 18,3400198 | 6,412754317 | 3,49659073 |
| UDP-glucose 4-epimerase;UDP-glucose 4-epimerase (Fragment);UDP-glucose 4-epimerase (Fragment);UDP-glucose 4-epimerase (Fragment) | GALE | 1146,60375 | 401,4030139 | 3,50079977 |
| Heat shock protein family A (Hsp70) member 9;Stress-70 protein, mitochondrial;Heat shock protein family A (Hsp70) member 9;Stress-70 protein, mitochondrial;Heat shock 70kDa protein 9B (Mortalin-2), isoform CRA_a;Stress-70 protein, mitochondrial | HSPA9 | 590,560181 | 206,9356431 | 3,50405682 |
| Thioredoxin-like protein 1 | TXNL1 | 35,9420019 | 12,61841225 | 3,51077057 |
| Major histocompatibility complex, class II, DR beta 1 (Fragment);HLA class II histocompatibility antigen DR beta chain;HLA class II histocompatibility antigen, DR beta 3 chain | HLA-DRB1;HLA-DRB1;HLA-DRB3 | 93,7152954 | 32,99364008 | 3,52062488 |
| Acyl-CoA dehydrogenase family member 10 (Fragment);Acyl-CoA dehydrogenase family member 10;Acyl-CoA dehydrogenase family member 10 | ACAD10 | 1796,25929 | 639,9113746 | 3,56246661 |
| L-xylulose reductase (Fragment);L-xylulose reductase (Fragment);L-xylulose reductase (Fragment);L-xylulose reductase (Fragment);L-xylulose reductase (Fragment);L-xylulose reductase (Fragment);L-xylulose reductase | DCXR | 67,5884605 | 24,17326357 | 3,57653709 |
| Immunoglobulin lambda variable 2-18 | IGLV2-18 | 171,424907 | 61,35760838 | 3,57927033 |
| Immunoglobulin heavy variable 3-7 | IGHV3-7 | 1007,64541 | 361,9111264 | 3,59165161 |
| Prostaglandin E synthase 3 | PTGES3 | 38,8952705 | 13,97119562 | 3,59200372 |
| Peptidase, mitochondrial processing subunit alpha | PMPCA | 78,1945099 | 28,14125029 | 3,59887802 |
| Calreticulin | CALR | 49,83619 | 17,98022497 | 3,60786508 |
| Retinol-binding protein 4;Retinol-binding protein | RBP4 | 224,817224 | 81,65578232 | 3,63209637 |
| Sentrin-specific protease 8 | SENP8 | 8,36944208 | 3,056004056 | 3,65138324 |
| Tubulin beta 4A class IVa;Tubulin beta chain;Tubulin beta chain;Tubulin beta 4A class IVa;Tubulin beta-4A chain | TUBB4A | 27,8353733 | 10,23504825 | 3,67699335 |
| Immunoglobulin lambda variable 3-21 | IGLV3-21 | 385,070935 | 145,4939175 | 3,77836664 |
| Proteasome assembly chaperone 1 | PSMG1 | 142,233006 | 54,20341432 | 3,81088861 |
| Immunoglobulin heavy constant gamma 4 | IGHG4 | 3182,99346 | 1214,83725 | 3,81665016 |
| RAB2B, member RAS oncogene family;RAB2B, member RAS oncogene family;RAB2A, member RAS oncogene family;RAB2A, member RAS oncogene family (Fragment);Ras-related protein Rab-2A;Ras-related protein Rab-2B | RAB2B;RAB2B;RAB2A;RAB2A;RAB2A;RAB2B | 30,288768 | 11,5997821 | 3,82973058 |
| Titin | TTN | 2917,12222 | 1142,852186 | 3,91773844 |
| SEC14-like protein 4 | SEC14L4 | 70,9061447 | 27,94638362 | 3,94132042 |
| Tropomyosin 4;Tropomyosin 4;Tropomyosin alpha-4 chain | TPM4 | 33,7531734 | 13,31070896 | 3,94354297 |
| Secernin-2;Secernin-2;Secernin-2 (Fragment);Secernin-2;Secernin-2 | SCRN2 | 8,19501858 | 3,243179929 | 3,95750162 |
| SH3 domain-binding glutamic acid-rich-like protein 2 | SH3BGRL2 | 19,6747902 | 7,975523037 | 4,05367628 |
| Fatty acid binding protein 5;Fatty acid-binding protein 5 | FABP5 | 33,2904507 | 13,56707607 | 4,07536569 |
| Tropomodulin-1 | TMOD1 | 38,5590717 | 15,81189562 | 4,10069406 |
| Rho GDP-dissociation inhibitor 1;Rho GDP-dissociation inhibitor 1 (Fragment);Rho GDP-dissociation inhibitor 1;Rho GDP-dissociation inhibitor 1 | ARHGDIA | 503,099011 | 208,6596868 | 4,14748752 |
| S-adenosylmethionine synthase isoform type-2 | MAT2A | 11,9090782 | 4,953327799 | 4,15928732 |
| CD5 antigen-like | CD5L | 275,268512 | 115,9327383 | 4,21162368 |
| Prenylcysteine oxidase 1 | PCYOX1 | 26,5553265 | 11,19007269 | 4,21387125 |
| Bridging integrator 3 | BIN3 | 57,9664661 | 24,8128979 | 4,28056074 |
| SAA2-SAA4 readthrough | SAA2-SAA4 | 193,868776 | 83,54906519 | 4,30956789 |
| Transmembrane protein 126A | TMEM126A | 323,412775 | 142,0064712 | 4,3908739 |
| Apolipoprotein B mRNA editing enzyme catalytic subunit 3B;DNA dC->dU-editing enzyme APOBEC-3A;DNA dC->dU-editing enzyme APOBEC-3B | APOBEC3B;APOBEC3A;APOBEC3B | 33,6446598 | 14,87578357 | 4,42143974 |
| Cystatin-B | CSTB | 220,113159 | 97,34106526 | 4,42231922 |
| Serine/threonine-protein phosphatase CPPED1 | CPPED1 | 186,826231 | 84,56887986 | 4,52660632 |
| Probable non-functional immunoglobulin kappa variable 2D-24;Immunoglobulin kappa variable 2-24 | IGKV2D-24;IGKV2-24 | 444,086517 | 201,3002297 | 4,53290568 |
| Schlafen family member 12;Schlafen family member 12;Schlafen family member 12;Schlafen family member 12;Schlafen family member 12;Ribonuclease SLFN12 | SLFN12 | 59,3501793 | 26,95771639 | 4,54214574 |
| Immunoglobulin heavy constant gamma 3 (G3m marker) (Fragment);Immunoglobulin heavy constant gamma 3 (G3m marker) (Fragment);Immunoglobulin heavy constant gamma 3 | IGHG3 | 1347,64478 | 612,5551233 | 4,54537525 |
| ATP-sensitive inward rectifier potassium channel 8 | KCNJ8 | 36,5735687 | 16,68862359 | 4,56302849 |
| Immunoglobulin heavy variable 3-13 | IGHV3-13 | 63,5553425 | 29,3056762 | 4,61104843 |
| Alcohol dehydrogenase class-3 | ADH5 | 73,4948403 | 34,8413825 | 4,74065694 |
| Immunoglobulin lambda variable 1-51 | IGLV1-51 | 131,453148 | 62,42274087 | 4,74866839 |
| Glutathione S-transferase Mu 3 | GSTM3 | 55,2379505 | 26,35757191 | 4,7716419 |
| Stathmin | STMN1 | 5,88610692 | 2,817150703 | 4,78610182 |
| Estrogen related receptor alpha;Estrogen related receptor alpha (Fragment);Steroid hormone receptor ERR1 | ESRRA | 311,268759 | 149,0871019 | 4,78965838 |
| Apolipoprotein E | APOE | 1552,86327 | 747,831002 | 4,81582002 |
| Immunoglobulin heavy constant mu | IGHM | 37194,0289 | 18342,16366 | 4,93148073 |
| Heat shock protein family B (small) member 1;Heat shock protein beta-1;Heat shock protein beta-1 | HSPB1 | 303,13405 | 149,780182 | 4,94105436 |
| Farnesyl pyrophosphate synthase (Fragment);(2E,6E)-farnesyl diphosphate synthase (Fragment);Farnesyl pyrophosphate synthase | FDPS | 39,564901 | 19,70090739 | 4,97939004 |
| Hemoglobin subunit gamma-1 | HBG1 | 8603,7665 | 4355,896638 | 5,06277877 |
| Prefoldin subunit 1 | PFDN1 | 64,366272 | 32,60676739 | 5,06581574 |
| Phospholipid transfer protein | PLTP | 153,35808 | 77,81837394 | 5,0742924 |
| Complement factor H related 2;Complement factor H related 2 (Fragment);Complement factor H related 2;Complement factor H-related protein 2 | CFHR2 | 22,2764957 | 11,37661577 | 5,10700423 |
| Hemoglobin subunit gamma-2 | HBG2 | 922,436926 | 485,1130376 | 5,25903749 |
| Dopamine beta-hydroxylase | DBH | 32,2624538 | 17,03374619 | 5,27974292 |
| Immunoglobulin lambda variable 2-14 | IGLV2-14 | 43,7624405 | 23,63408988 | 5,40054202 |
| Protein adenylyltransferase SelO, mitochondrial | SELENOO | 6,78230376 | 3,73392436 | 5,5053924 |
| Apolipoprotein C-II | APOC4-APOC2;APOC2;APOC2 | 1637,87825 | 923,6986146 | 5,63960486 |
| Tetratricopeptide repeat domain 38 (Fragment);Tetratricopeptide repeat protein 38 | TTC38 | 17,1210779 | 9,707715857 | 5,67003778 |
| Tubulin--tyrosine ligase-like protein 12 | TTLL12 | 106,537704 | 61,52477558 | 5,77492974 |
| Uncharacterized protein (Fragment) |  | 594,686914 | 344,0348646 | 5,78514268 |
| Haptoglobin-related protein | HPR | 708,545923 | 422,4421541 | 5,96209985 |
| Apolipoprotein C-III | APOC3 | 2362,00547 | 1447,790328 | 6,12949608 |
| EGF containing fibulin extracellular matrix protein 1 (Fragment);EGF-containing fibulin-like extracellular matrix protein 1 | EFEMP1 | 26,2761498 | 17,98697587 | 6,84536205 |
| Immunoglobulin heavy constant delta (Fragment);Immunoglobulin heavy constant delta | IGHD | 519,209045 | 370,9061788 | 7,14367714 |
| 1-acylglycerol-3-phosphate O-acyltransferase 5;1-acyl-sn-glycerol-3-phosphate acyltransferase epsilon | AGPAT5 | 20,1601185 | 15,23813025 | 7,55855193 |
| Dermcidin | DCD | 37,1298939 | 32,37225126 | 8,71864901 |
| Immunoglobulin lambda variable 8-61 | IGLV8-61 | 97,3250816 | 85,07866191 | 8,74169952 |
| Probable non-functional immunoglobulin heavy variable 3-38 | IGHV3-38 | 47,2155853 | 42,3718277 | 8,97411891 |
| Immunoglobulin lambda-like polypeptide 1 | IGLL1 | 2162,52917 | 2036,640818 | 9,41786518 |

## Protocol for isoelectric focusing in the 1^st^ dimension

**Supplementary Table S11:** Protocol for isoelectric focusing of the IPG strip pH 3–10 in the IPGphore instrument.

| Step | Step/Grad | Step/Grad | Time |
| --- | --- | --- | --- |
| S1 | Stp | 30 V | 12 h |
| S2 | Stp | 200 V | 1 h |
| S3 | Stp | 500 V | 1 h |
| S4 | Stp | 1 000 V | 1 h |
| S5 | Grd | 8 000 V | 30 min |
| S6 | Stp | 8 000 V | 4 h 30 min |

## Protocol for SDS-PAGE in the 2^nd^ dimension

**Supplementary Table S12:** Protocol for SDS-PAGE on Serva HPE BlueTower using a 2D HPE Large Gel NF 10–15 %.

| Step | Voltage | Current | Power | Time |
| --- | --- | --- | --- | --- |
| S1 | 100 V | 7 mA | 4 W | 30 min |
| S2 | 200 V | 13 mA | 12 W | 30 min |
| S3 | 300 V | 20 mA | 20 W | 10 min |
| S4 | 1 000 V | 40 mA | 120 W | 3 h 5 min |
